# RNA Sequencing of the Pituitary Gland and Association Analyses Reveal *PRKG2* as a Candidate Gene for Growth and Carcass Traits in Chinese Ningdu Yellow Chickens

**DOI:** 10.3389/fvets.2022.892024

**Published:** 2022-06-16

**Authors:** Xinwei Xiong, Min Zhou, Xuenong Zhu, Yuwen Tan, Zhangfeng Wang, Jishang Gong, Jiguo Xu, Yafang Wen, Jianxiang Liu, Xutang Tu, Yousheng Rao

**Affiliations:** ^1^Institute of Biological Technology, Nanchang Normal University, Nanchang, China; ^2^Key Laboratory for Genetic Improvement of Indigenous Chicken Breeds of Jiangxi Province, Nanchang, China

**Keywords:** Ningdu yellow chickens, growth and carcass traits, RNA-seq, candidate gene, *PRKG2* gene

## Abstract

Growth and carcass traits are of great economic importance to the chicken industry. The candidate genes and mutations associated with growth and carcass traits can be utilized to improve chicken growth. Therefore, the identification of these genes and mutations is greatly importance. In this study, a total of 17 traits related to growth and carcass were measured in 399 Chinese Ningdu yellow chickens. RNA sequencing (RNA-seq) was performed to detect candidate genes using 12 pituitary gland samples (six per group), which exhibited extreme growth and carcass phenotypes: either a high live weight and carcass weight (H group) or a low live weight and carcass weight (L group). A differential expression analysis, utilizing RNA-seq, between the H and L groups identified 428 differentially expressed genes (DEGs), including 110 up-regulated genes and 318 down-regulated genes. Gene ontology (GO) and Kyoto Encyclopedia of Genes and Genomes (KEGG) enrichment analyses of the identified genes showed a significant enrichment of 158 GO terms and two KEGG pathways, including response to stimulus and neuroactive ligand-receptor interaction, respectively. Furthermore, RNA-seq data, qRT–PCR, and quantitative trait transcript (QTT) analysis results suggest that the *PRKG2* gene is an important candidate gene for growth and carcass traits of Chinese Ningdu yellow chickens. More specifically, association analyses of a single nucleotide polymorphism (SNP) in *PRKG2* and growth and carcass traits showed that the SNP *rs16400745* was significantly associated with 12 growth and carcass traits (*P* < 0.05), such as carcass weight (*P* = 9.68E-06), eviscerated weight (*P* = 3.04E-05), and semi-eviscerated weight (*P* = 2.14E-04). Collectively, these results provide novel insights into the genetic basis of growth in Chinese Ningdu yellow chickens and the SNP *rs16400745* reported here could be incorporated into the selection programs involving this breed.

## Introduction

Growth and carcass traits are of great economic importance to the chicken industry. The most valuable growth and carcass traits include body weight, carcass weight, eviscerated weight, and semi-eviscerated weight. All growth and carcass traits are complex because they are affected by multiple interacting factors, including genetic background (e.g., species, major genes, and gene interactions) and environmental background (e.g., feeding, management, and slaughter conditions). Additionally, most growth and carcass traits have been genetically improved through estimated breeding value or phenotypic selection in the past decades (1, 2), which reduces the effectiveness of traditional phenotype-based breeding strategies that rely solely on the phenotypes of relatives. Moreover, the phenotypes of carcass traits can only be recorded after slaughter, which precludes the selection of breeding individuals based on these traits (1). Therefore, it is very important to elucidate the genetic mechanisms underlying these traits and identify candidate genes for traits related to growth and carcass in chickens.

In recent years, with the rapid development and reduced costs of next generation sequencing (NGS), RNA sequencing (RNA-seq) has exhibited great potential in providing more accurate and comprehensive information regarding changes in gene expression between different conditions or phenotypes (3–7). Substantial studies have used RNA-seq to identify differentially expressed genes (DEGs) for eggshell quality, feed efficiency and sex differentiation in chicken (3, 8–10). For example, Ayers et al. used RNA-seq to produce a comprehensive profile of gene expression in chicken blastoderms and embryonic gonads prior to sexual differentiation (9), and they found significant sexually dimorphic gene expression in both tissues pre-dating gonadogenesis and comprehensively annotated the W-chromosome. Using RNA-seq and association analyses, Chen et al. found that *FOXO3* is a candidate gene for growth traits in chickens (11). Through high-throughput RNA sequencing, Zhao et al. identified 154 DEGs and the cognate biological pathways affecting the carcass traits (12). Therefore, a comprehensive gene expression study using RNA-seq is required for better understanding the molecular basis underlying the growth and carcass traits in Chinese Ningdu yellow chickens.

Ningdu yellow chicken is a local Chinese breed and has enjoyed a positive culinary reputation for a long time (13). In this study, to identify the genes related to growth and carcass traits, a differential expression analysis was performed on pituitary glands harvested from Chinese Ningdu yellow chickens with either a high live weight and carcass weight (H group) or a low live weight and carcass weight (L group) using RNA-seq. After RNA-seq analysis, we performed qPCR to validate the data and QTT analysis, and identified *PRKG2* as a candidate gene for growth and carcass traits. Furthermore, we assessed SNPs in the chicken *PRKG2* gene and analyzed the associations between *PRKG2* gene polymorphisms and growth and carcass traits in Chinese Ningdu yellow chickens. These results will help to identify the genes underlying growth and carcass traits and provide a theoretical reference for the molecular-assisted breeding of desirable chickens.

## Materials and Methods

### Experimental Population, Tissue Preparation, and Phenotype Measurement

The Ningdu yellow population used in this study comprised 399 purebred male chickens. All of the chickens were born and raised for 1 day by Jiangxi Nanshi Science and Technology Co., Ltd (Nanchang, ChinaNanchang, China). They were then randomly selected from the same batch and transferred to a farm in the city of Nanchang, ChinaNanchang, China. The chickens were fed with the same diet under a standardized feeding and management regimen, and given *ad libitum* access to water. At the age of 16 weeks, all of chickens were slaughtered at a commercial abattoir in Nanchang. After the Ningdu yellow chickens were slaughtered, the pituitary gland was carefully harvested for RNA isolation. The tissues were put into the sterile and frozen cryopreservation tubes and dipped into liquid nitrogen, and then conserved in −80°C ultra-freezer until RNA extraction. Moreover, a total of 17 growth and carcass traits were measured using “The Poultry Production Performance Terms and Measurement Statistics Method (NY/T823-2004),” including body length, live weight, carcass weight, carcass rate, semi-eviscerated weight, semi-eviscerated rate, eviscerated weight, eviscerated rate, breast muscle weight, breast muscle rate, chest circumference, average daily gain, chest depth, birth weight, back width, chest angle, and chest width. Each trait was measured by the same researcher.

### Total RNA Isolation, Library Construction, and Sequencing

A total of 12 pituitary glands of Chinese Ningdu yellow chickens exhibiting extreme growth and carcass phenotypes, with either a high live weight and carcass weight (H group) or a low live weight and carcass weight (L group) (Supplementary Table S1), were randomly divided into H and L groups with six chickens per group. The total RNA was isolated with TRIzol (Invitrogen, USA) by following the manufacture's instruction. The residual DNA was cleared away from total RNA by incubation with RNase-free DNase I (New England Biolabs, UK) at 37°C for 30 min. The quality of total RNA was assessed by a 2100 Bioanalyzer (Agilent, UK) and agarose gel electrophoresis. Three μg RNA per sample was used as input material for RNA preparation. Finally, 12 samples with RNA integrity number (RIN) values above eight were used for libraries construction. Sequencing libraries were generated using NEBNext® Ultra™ RNA Library Prep Kit for Illumina® (NEB, USA) by following manufacturer's recommendations, and index codes were added to attribute sequences to each sample. Briefly, mRNA was purified from total RNA using poly-T oligo-attached magnetic beads. Fragmentation was carried out using divalent cations under elevated temperature in NEBNext First Strand Synthesis Reaction Buffer. First strand cDNA was synthesized using random hexamer primer and M-MuLV Reverse Transcriptase (NaseH–). Subsequently, second strand cDNA synthesis was performed using DNA Polymerase I and RNase H. The remaining overhangs were converted into blunt ends *via* exonuclease/polymerase activities. After adenylation of 3′ ends of DNA fragments, NEBNext Adaptor with hairpin loop structure was ligated to prepare for hybridization. In order to select cDNA fragments of preferential ~200 bp in length, the library fragments were purified with AMPure XP system (Beckman Coulter, Beverly, USA). Then 3 μl USER Enzyme (NEB, USA) was used with size-selected, adaptor-ligated cDNA at 37°C for 15 min, followed by 5 min at 95°C before PCR. Then PCR was performed with Phusion High-Fidelity DNA polymerase, Universal PCR primers and Index (X) Primer. At last, PCR products were purified (AMPure XP system) and library quality was assessed using the Agilent Bioanalyzer 2100 system. The clustering of the index-coded samples was performed on a cBot Cluster Generation System using TruSeq PE Cluster Kit v3-cBot-HS (Illumia) according to the manufacturer's instructions. After cluster generation, the library preparations were sequenced on an Illumina Novaseq platform and 150 bp paired-end reads were generated.

### Sequence Reads Mapping, Assembly and Gene Expression Quantitation

The raw reads with N base contents more than 10% in a read and the percentage of low quality (Q ≤ 5) bases more than 50% were removed (14). The chicken reference genome Gallus_gallus-6.0/galGal6 was downloaded from genome website (http://asia.ensembl.org/Gallus_gallus/Info/Index) directly. The clean reads were mapped with TopHat (15) version 2.0.13. TopHat incorporates the Bowtie2 version 2.2.5 algorithm to perform the alignment (16). The aligned read files were processed by Cufflinks (17) version 2.2.1. Cufflinks uses the normalized RNA-Seq fragment counts to measure the relative abundances of transcripts. The unit of measurement is Fragments Per Kilobase of exon per Million fragments mapped (FPKM). Confidence intervals for FPKM estimates were calculated using a Bayesian inference method (18). Cuffdiff, a part of the Cufflinks package, takes the aligned reads from two or more conditions and reports genes and transcripts that are differentially expressed using a rigorous statistical analysis (19). Partial Least Squares-Discriminant analysis (PLS-DA) was performed to evaluate the whole gene transcripts between the H and L groups (20). Finally, Genes with a false discovery rate of <5% (i.e., FDR <0.05) and the difference ratio of FPKM between the H and L groups no less than two (|log2 fold change|≥ 1.0) as the significant threshold to identify differential expression.

### GO Enrichment Analysis and KEGG Pathway Analysis of DEGs

Gene Ontology (GO) enrichment analysis and KEGG pathway analysis of the DEGs were implemented using DAVID Bioinformatics Resources (2021 Update) (21). Hypergeometric test was applied to map all DEGs to terms in GO (http://www.geneontology.org/GO.database.shtml) and KEGG pathway (http://www.genome.jp/kegg/pathway.html) database and search significantly enriched GO terms and KEGG pathway in DEGs comparing to the genome background. The FDR <0.05 was taken as a threshold of significance for GO terms and KEGG pathway.

### Complementary DNA (cDNA) Synthesis and Quantitative Reverse Transcription Polymerase Chain Reaction (qRT-PCR) Analysis

cDNA was synthesized from 1 μg of total RNA using a PrimerScript RT reagent Kit (Takara, Japan) by following the standard manual. qRT-PCR was performed in a 10 μl reaction system containing 1 μl of 2.5-fold diluted cDNA, 5 μl of Power SYBR Green PCR Master Mix (Applied Biosystems, USA), 2 pM of each forward and reverse primer (Supplementary Table S2), and 3.6 μl water. PCR was conducted on a Bio-Rad CFX96 instrument (Bio-Rad) under the following cycling conditions: 10 min at 95°C, followed by 40 cycles at 95°C for 15 s and 60°C for 50 sec. *GAPDH* was used as the endogenous control. The quantification of gene expression levels was performed using the comparative Ct (2^−ΔΔ*Ct*^) method. All assays were performed in triplicate and the average values were calculated.

### Polymorphisms in *PRKG2* and Statistical Analysis

Primer (Supplementary Table S2) was designed using Primer 5.0 software based on chicken *PRKG2* gene sequences (SSC4: 45635577 – 45636527) and synthesized by GENERAY Biotech Co., Ltd (Shanghai, China). PCR was performed in a 30 μl reaction mixture containing 1 μl of DNA, 1 μl of forward and reverse primers (10 μM), 15 μl of 2 × Taq PCR MasterMix, and 12 μl ddH_2_O. All amplifications included an initial denaturing step of 3 min at 95°C, followed by 35 cycles of 20 s at 94°C, 20 s at an optimized annealing temperature and 40 s at 72°C, with a final extension of 5 min at 72°C. The PCR products were sequenced by the GENERAY Biotech Co., Ltd (Shanghai, China). The ABI 3730xl DNA Sequencer was used for sequencing by the Sanger method. Long fragments were sequenced by bi-directional sequencing and then assembled using DNAStar software. SNPs were identified by comparative analysis of the complete *PRKG2* sequence using Seqman software. Association analysis between the SNP and phenotypic traits was performed using the general linear model procedure in Plink v 1.07 (22). The slaughter batch was fitted as fixed effect in the model.

The associations between gene expression levels and the phenotypic values of growth and carcass traits were evaluated with Pearson correlation coefficient by using the *cor* function in the R software program. According to the previously reported QTT analyses in *Drosophila melanogaster* (23), pig (24) and human obesity (25), a conservative *P* < 0.0005 was set as the significance threshold.

## Results

### Summary Statistics and Correlation Analysis for Growth and Carcass Traits

In this study, 399 Ningdu yellow chickens were phenotyped across 17 growth and carcass traits. The summary statistic results for the 17 traits are shown in Table 1. Results indicated that most of the tested growth and carcass traits had low coefficients of variation, with the highest value of 25.01% for breast muscle weight and the lowest value of 5.86 for body length.

**Table 1:** Summary statistics of growth and carcass traits from Chinese Ningdu yellow chicken population.

**Traits**	**Mean**	**S.D.^**a**^**	**Min**	**Max**.	**C.V^**b**^ (%)**
Carcass weight (g)	758.51	121.93	401.40	1139.30	16.07
Eviscerated weight (g)	524.89	90.39	270.20	848.50	17.22
Semi-eviscerated weight (g)	668.69	109.50	361.00	1034.00	16.38
Breast muscle weight (g)	57.45	14.37	20.80	104.60	25.01
Carcass rate (%)	82.92	8.12	45.84	92.39	9.79
Eviscerated rate (%)	57.28	6.00	31.24	66.53	10.48
Semi-eviscerated rate (%)	73.02	7.35	41.29	84.89	10.06
Breast muscle rate (%)	6.24	1.20	2.30	10.20	19.15
Live weight (g)	915.65	122.23	566.40	1275.3	13.35
Body length (cm)	17.65	1.03	14.80	23.10	5.86
Chest circumference (cm)	19.77	1.61	14.40	25.20	8.13
Average daily gain (g)	7.88	1.07	4.84	10.43	13.55
Chest depth (mm)	79.21	6.58	54.41	102.89	8.31
Birth weight (g)	29.99	3.83	17.80	40.80	12.78
Back width (mm)	59.75	6.66	42.12	80.46	11.15
Chest angle (°)	50.11	5.70	35.44	69.50	11.37
Chest width (mm)	53.05	8.91	34.53	74.60	16.80

^a^*Standard deviation*. ^b^*Coefficient of Variation*.

**Figure 1:**
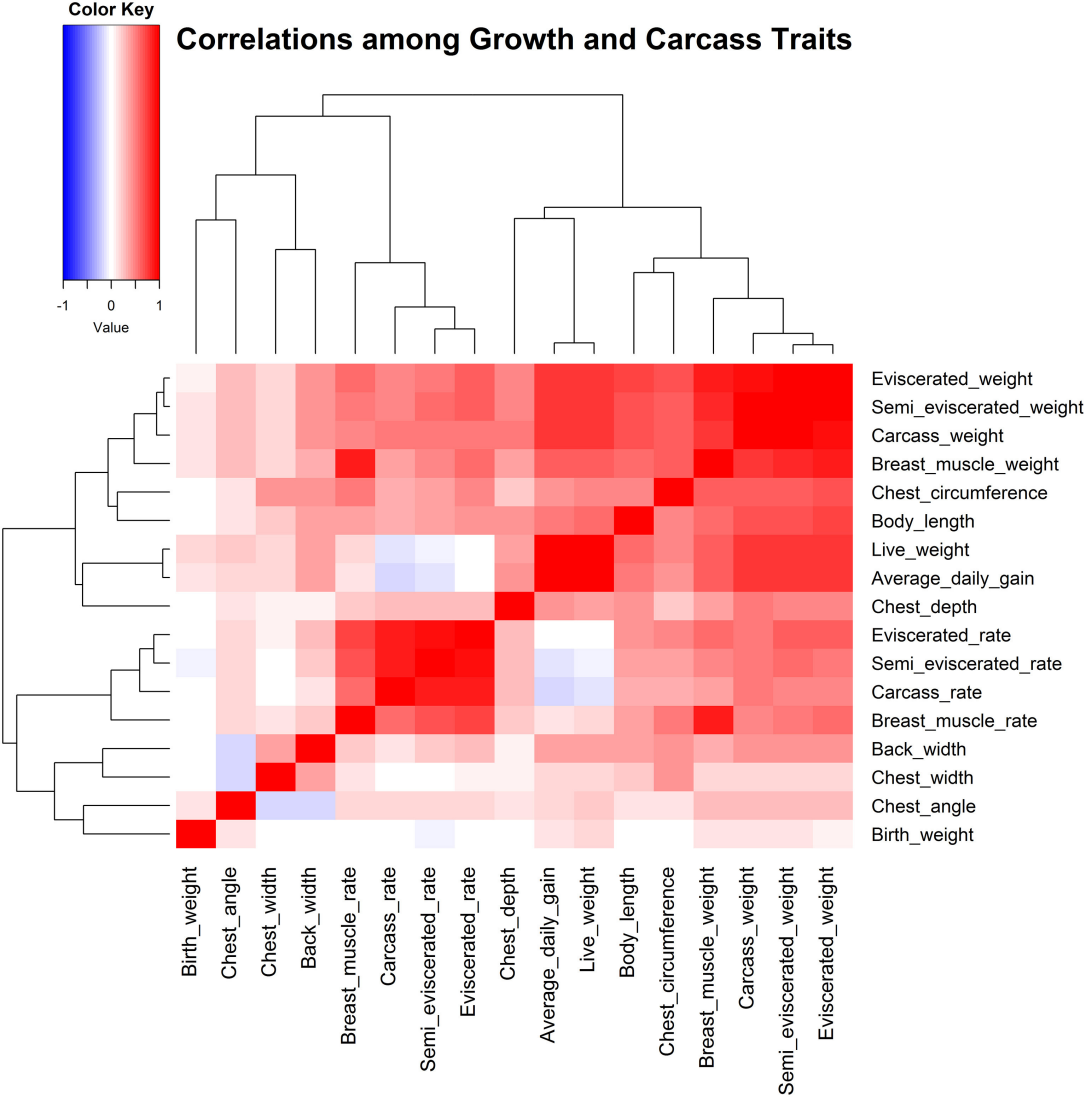
Correlation analysis among 17 traits related to growth and carcass.

Correlational analyses were then conducted among 17 traits for growth and carcass traits (Figure 1, Supplementary Tables S3, S4). Results indicated that most growth and carcass traits had high correlations. More specifically, the carcass weight, semi-eviscerated weight, and breast muscle weight were all significantly, positively correlated with other growth and carcass traits. For example, the carcass weight was significantly, positively correlated with all other growth and carcass traits, especially for semi-eviscerated weight and eviscerated weight (*r* values were 0.95; *P* < 0.001).

### Differential Gene Expression in Pituitary Gland Tissue

To identify the genes associated with growth and carcass traits, genome-wide expression levels of genes in the pituitary gland were determined by RNA-seq for 12 Chinese Ningdu yellow chickens. After the adaptor sequences, ambiguous nucleotides, and low-quality sequences were removed, a total of 543,865,996 (81.60 Gb of data bulk) clean reads were generated from the 12 libraries and were divided into two groups with either a high live weight and carcass weight (H group) or a low live weight and carcass weight (L group). The summary of reads and the mapped genomic features are depicted in Supplementary Table S5. The results showed that about 85.59–90.94% of the clean reads mapped to the *Gallus gallus* reference genome, of which 1.35–1.75% have multiple alignments.

**Figure 2:**
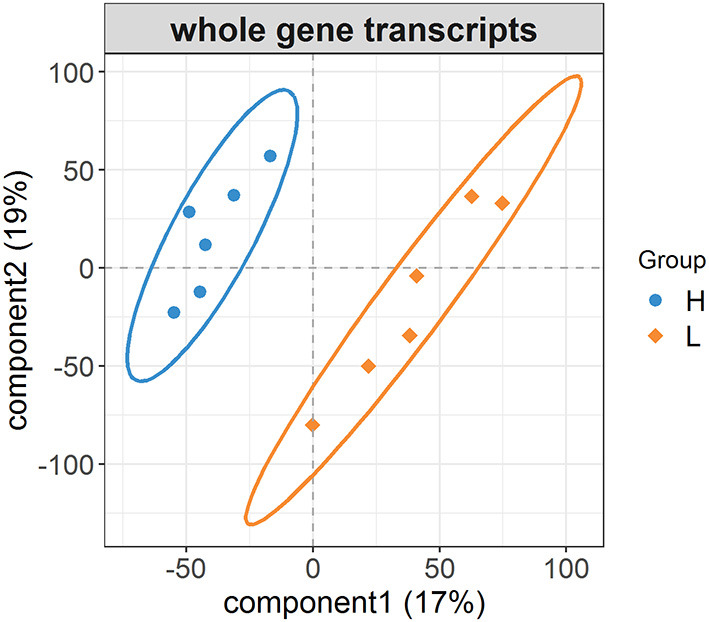
The changes in whole gene transcripts between the H and L groups. PLS-DA plot of whole gene transcripts indicating the significant differentiation of gene expression profiles between the H and L groups.

**Figure 3:**
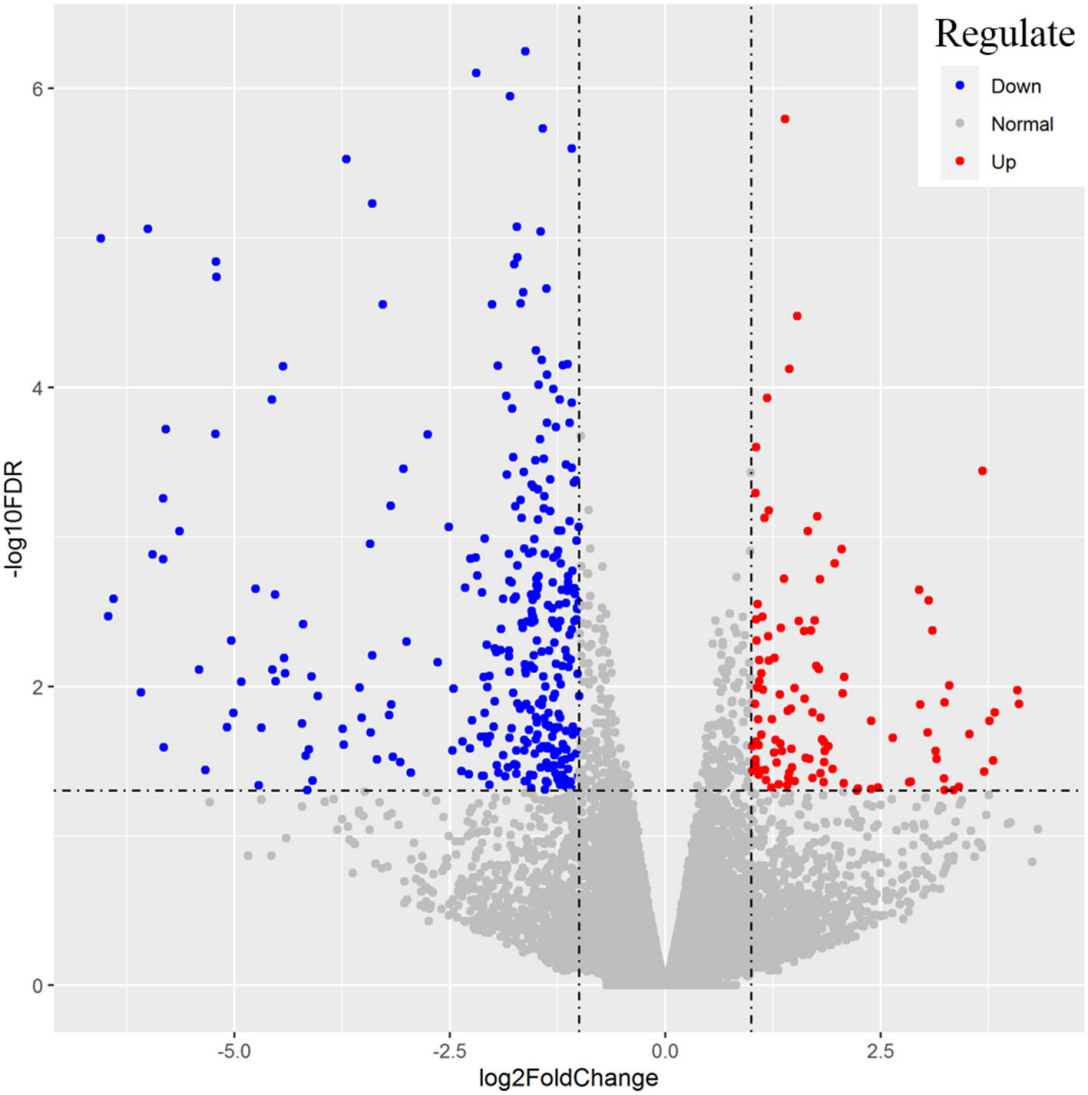
Representation of the differentially expressed genes between the H and L groups. The x-axis values are the log2 (fold change) and the y-axis values are –log10 FDR. Genes with higher expression levels in H group compared with L group were denoted as “up-regulated” and indicated in red, while those with lower expression levels in H group were “down-regulated” and indicated in blue.

After the repeated and short-length sequences were eliminated, a total of 21,380 gene transcripts were aligned to the reference genome. An obvious shift in the whole gene transcripts was observed between the H and L groups (Figure 2). Specifically, the differential gene expression analysis identified a total of 428 (|log2 fold change| ≥ 1.0, FDR <0.05) DEGs between the H and L groups. In this study, the DEGs with higher expression levels in the H group as compared to the L group were denoted as “up-regulated,” while those with lower expression levels in the H group as compared to the L group were denoted as “down-regulated.” The expression levels of 110 among 428 genes were determined to be up-regulated in the H group, while the other 318 genes were down-regulated (Figure 3, Supplementary Table S6). For example, *SLCO1C1* (log2 fold change = 3.68, FDR = 3.62E-04), *EGR4* (log2 fold change = 3.30, FDR = 9.86E-03), and *RLN3* (log2 fold change = 2.07, FDR = 4.46E-02) were found to be significantly upregulated; and *MC1R* (log2 fold change = −4.53, FDR = 2.43E-03), *MYB* (log2 fold change = −3.43, FDR = 1.11E-03), and *IRF4* (log2 fold change = −3.40, FDR = 5.88E-06) were found to be significantly downregulated.

### GO Enrichment Analysis and KEGG Pathway Analysis of DEGs

**Figure 4:**
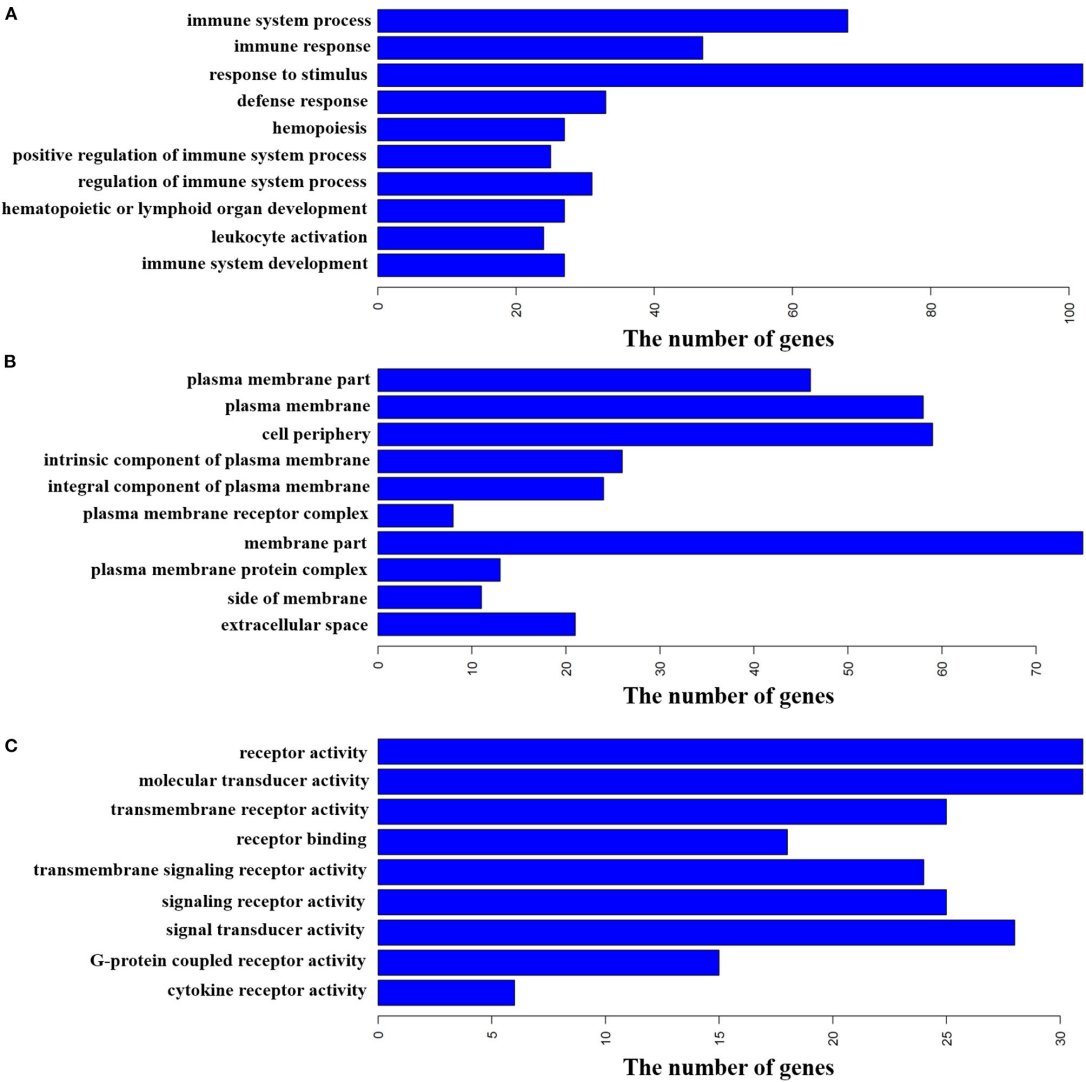
Functional annotation of DEGs by gene ontology (GO) analysis. The bar plot represents the gene counts within each GO category. All function or process terms listed have enrichment of FDR <0.05. **(A)** Biological process (top 10). **(B)** Cellular component (top 10). **(C)** Molecular function.

The NCBI web-based functional annotation tool DAVID Bioinformatics Resources (2021 Update) was used to investigate the functional associations of the changes in gene expression identified in the pituitary glands between the two groups (21, 26). GO biological process, molecular function, and cellular component were selected as the annotation categories for clustering. The software identifies enriched ontologies for a particular gene list and clusters statistically significant terms for their constituent genes. The gene list used in this analysis contained the 428 DEGs. The results showed that these DEGs were enriched (FDR <0.05) in many more GO terms than expected (Supplementary Table S7), including 134 terms significantly enriched in GO biological process and 15 terms significantly enriched in GO cellular component. For example, immune system process, immune response, and response to stimulus were enriched in GO biological process (Figure 4A), and plasma membrane part, plasma membrane, and cell periphery were enriched in GO cellular component (Figure 4B). Specifically, nine terms were significantly enriched in GO molecular function. Such as, receptor activity, molecular transducer activity, and transmembrane receptor activity (Figure 4C).

Furthermore, the KEGG Pathway Database, which documents many types of influences and interactions, was used to help us generate an overview of the network pathway. The results showed that neuroactive ligand-receptor interaction including 14 genes, FDR = 6.00E-03, and cytokine-cytokine receptor interaction including 10 genes, FDR = 8.10E-03 were the only two terms significantly enriched in the KEGG pathway. The DEGs enriched in two KEGG pathways are shown in Supplementary Table S8. Specifically, the differentially expressed *GH* (growth hormone) gene and *GHRHR* (growth hormone releasing hormone receptor) gene, which are related to growth, were included in neuroactive ligand-receptor interaction pathway.

### Validation of DEGs in RNA-Seq

**Figure 5:**
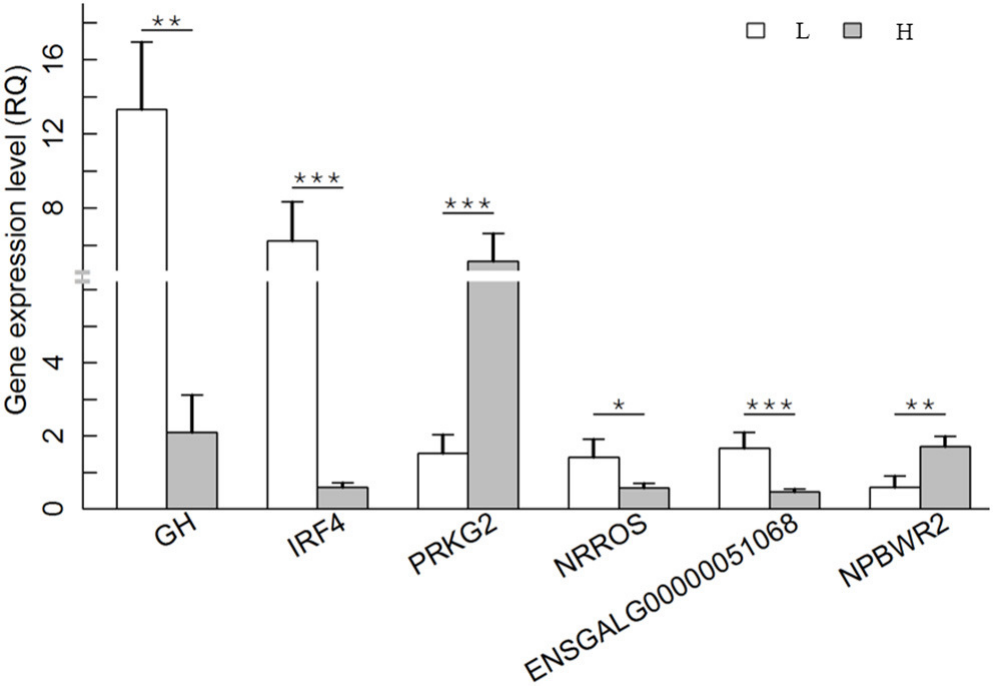
Differential expression levels of the following DEGs between H and L groups was confirmed by qRT–PCR. The x-axis indicates the DEGs, and the y-axis shows the relative quantity (RQ) value. The *P* value of each pair of comparison is presented in the figure. *P < 0.05; **P < 0.01; ***P < 0.001. H group: gray bars; L group: white bars.

To further validate the DEGs result, the expression patterns of six randomly selected DEGs were quantified by qRT–PCR. The six genes were *GH, IRF4* (interferon regulatory factor 4), *NPBWR2* (neuropeptides B and W receptor 2), *NRROS* (negative regulator of reactive oxygen species), *PRKG2* (protein kinase cGMP-dependent 2), and *ENSGALG00000051068*. A total of 12 RNA samples (six for each group) used for RNA sequencing analysis, were also used for qRT–PCR analysis. The results showed that all six genes were differentially expressed between the H and L groups, supporting a high reproducibility of RNA-seq results (Figure 5).

### Investigation of Candidate Genes by Quantitative Trait Transcript (QTT) Analysis

To identify the candidate genes for chicken growth and carcass traits from the DEGs, the QTT analysis was performed to test the correlation between gene expression levels and live weight or carcass weight values. Seven DEGs were identified to be significantly associated with live weight and three were identified to be significantly associated with carcass weight (Table 2). For example, two were positively correlated (*GRIA1* (*r* = 0.92, *P* = 1.81E-05) and *PRKG2* (*r* = 0.85, *P* = 4.82E-04)) and five were negatively correlated [i.e., *NRROS* (*r* = −0.91, *P* = 5.00E-05) and *IRF4* (*r* = −0.85, *P* = 4.31E-04)] with live weight. Furthermore, the eight DEGs were further investigated to determine if growth-related phenotypes or diseases had been reported in their corresponding knockout mice using the Mouse Genome Informatics database (http://www.informatics.jax.org/). For three of the candidate genes, *GRIA1, IRF4*, and *PRKG2*, the results supported a role for these genes in growth and carcass traits (Table 2).

**Table 2:** Candidate genes identified in pituitary gland for growth and carcass traits.

**Traits**	**Gene name**	**Chromosomes^**a**^**	**Position, bp^**a**^**	***R*-Value^**b**^**	***P*-Value**	**Phenotype in Knockout Mice^**c**^**
Live weight						
	ENSGALG00000051068	4	91264001–91278824	−0.92	1.81E-05	–
	GRIA1	13	13030976–13139199	0.92	1.81E-05	Decreased body size
	NRROS	9	4855404–4859977	−0.91	5.00E-05	–
	ARHGAP45	28	3019600–3031885	−0.90	5.99E-05	–
	SASH3	4	1634846–1637992	−0.88	1.79E-04	–
	IRF4	2	66457756–66470687	−0.85	4.31E-04	Increased body weight
	PRKG2	4	45615049–45636930	0.85	4.82E-04	Decreased body weight, decreased body length
Carcass weight						
	GRIA1	13	13030976–13139199	0.91	3.85E-05	Decreased body size
	ENSGALG00000045940	3	105078987–105090249	−0.85	4.46E-04	–
	PRKG2	4	45615049–45636930	0.85	4.98E-04	Decreased body weight, decreased body length

^a^*Chromosomal location of DEGs according to Gallus gallus genome reference assembly; ^b^The coefficient between gene expression and phenotype value; ^c^Phenotype in knockout mice were found in MGI (http://www.informatics.jax.org/)*.

### Sequencing of Variants in *PRKG2* Gene and Association Analysis Between SNPs and Growth and Carcass Traits

We next investigated the variants of the three candidate genes through RNA-seq data using GATK (v4.1.1.0) software. A total of 39 polymorphisms (35 SNPs and four indels) were identified in the 12 Chinese Ningdu yellow chickens. Of these mutations, the genotypes of the SNP *rs16400745* was the only SNP consistent with the genotypic patterns of the phenotypic values of the aforementioned 12 chickens, which was considered to the candidate SNP. The SNP *rs16400745* was 3 prime UTR variant in the *PRKG2* gene. We further genotyped the SNP *rs16400745* in a total of 399 Chinese Ningdu yellow chickens using the Sanger method (Primer: FP: TAAAGACTCCGAAACTCACT, RP: ACGCACCATAGACTCATT). The results showed that the SNP *rs16400745* had three genotypes. The frequencies of CC, CT, and TT in *rs16400745* were 0.40, 0.50, and 0.10, respectively.

An association analysis between the SNP *rs16400745* and 17 growth and carcass traits was performed, and the results showed that the SNP *rs16400745* was significantly associated with carcass weight (*P* = 9.68E-06), eviscerated weight (*P* = 3.04E-05), semi-eviscerated weight (*P* = 2.14E-04), breast muscle weight (*P* = 2.81E-04), live weight (*P* = 4.34E-04), body length (*P* = 4.95E-03), chest circumference (*P* = 8.33E-03), and average daily gain (*P* = 8.80E-03). The effects of the SNP *rs16400745* on growth and carcass traits are illustrated in Table 3. For example, the SNP *rs16400745* (T allele) was associated with an increased carcass weight and live weight by 37.50 and 33.00 g, respectively.

**Table 3:** The effects of the SNP *rs16400745* on traits related to growth and carcass.

**Traits**	**CC^**a**^**	**CT^**a**^**	**TT^**a**^**	**Effects^**b**^**	***P-*value**
	**(*n* = 159)**	**(*n* = 201)**	**(*n* = 39)**		
Carcass weight (g)	727.00 ± 9.45	776.00 ± 8.43	802.00 ± 19.08	37.50	9.68E-06
Eviscerated weight (g)	503.00 ± 7.10	536.00 ± 6.29	559.00 ± 14.21	28.00	3.04E-05
Semi-eviscerated weight (g)	644.00 ± 8.64	682.00 ± 7.65	699.00 ± 17.28	27.50	2.14E-04
Breast muscle weight (g)	54.50 ± 1.14	58.90 ± 1.01	62.30 ± 2.27	3.90	2.81E-04
Live weight (g)	891.00 ± 9.58	927.00 ± 8.52	957.00 ± 19.34	33.00	4.34E-04
Body length (cm)	17.50 ± 0.08	17.70 ± 0.07	17.90 ± 0.16	0.20	4.95E-03
Chest circumference (cm)	19.50 ± 0.13	19.90 ± 0.11	20.20 ± 0.26	0.35	8.33E-03
Average daily gain (g)	7.70 ± 0.09	7.96 ± 0.08	8.15 ± 0.18	0.23	8.80E-03
Breast muscle rate (%)	6.08 ± 0.09	6.33 ± 0.09	6.50 ± 0.19	0.21	0.017
Eviscerated rate (%)	56.40 ± 0.48	57.70 ± 0.42	58.40 ± 0.96	1.00	0.018
Chest depth (mm)	78.20 ± 0.52	79.90 ± 0.46	79.70 ± 1.05	0.75	0.036
Carcass rate (%)	81.90 ± 0.64	83.60 ± 0.57	83.90 ± 1.30	1.00	0.047
Birth weight (g)	29.50 ± 0.33	30.30 ± 0.29	30.30 ± 0.65	0.40	0.076
Semi-eviscerated rate (%)	72.20 ± 0.59	73.60 ± 0.52	73.20 ± 1.18	0.50	0.16
Back width (mm)	59.20 ± 0.53	60.10 ± 0.47	60.20 ± 1.07	0.50	0.24
Chest angle (°)	49.80 ± 0.45	50.40 ± 0.40	50.00 ± 0.91	0.10	0.53
Chest width (mm)	53.00 ± 0.71	53.00 ± 0.63	53.30 ± 1.43	0.15	0.90

^a^*The phenotypic was calculated by lsmeans package in the R environment. All of the traits are shown as the least square (LS) mean ± standard error (SE) of each genotype*. ^b^*Additive effects: (TT-CC)/2. The effects that allele “T” increases phenotype value in Ningdu yellow chickens*.

## Discussion

In this study, we used outbreed Ningdu yellow chickens, which show high correlations of growth and carcass traits. The transcriptional profiles of the pituitary gland, an organ that is highly related to chicken growth, were investigated using RNA-seq. The genes with differential expression between the H and L groups were identified. Many DEGs were found to be significantly upregulated or downregulated when comparing the H group to the L group. Three of the identified genes have been previously reported to have growth phenotypes in their corresponding knockout mice, which suggests they may be important candidate genes. Additionally, the associations between SNP in the *PRKG2* gene and growth and carcass traits were analyzed.

A total of 428 DEGs between the H and L groups were identified. The 428 genes were found to be enriched for 158 GO terms and two KEGG pathways, including a significant enrichment in immune system process, immune response, and response to stimulus associated with chicken growth. Some key genes in these groups may play crucial roles in chicken growth. For example, *GH* known as a major pituitary hormone, a critical regulator of organism growth and metabolism (27), and an important gene for growth and reproduction in poultry (28–31), is a member of the immune system process, immune response, and response to stimulus GO terms. Additionally, a total of 102 DEGs were enriched in response to stimulus. Of these DEGs, with the exception of *GH, GHRHR* is also highly expressed in pituitary gland, which implies a physiological or pathophysiological role, in addition to its normal endocrine function (32), that may affect the growth and development of animals (33). Additional genes, such as prolactin releasing hormone (*PRLH*) and relaxin 3 (*RLN3*) were also reported in the Mouse Genome Informatics database to be associated with growth in knockout mice. For example, the *PRLH* knockout mouse was associated with an increased body weight (34, 35), and the *RLN3* knockout mouse was associated with a decreased body weight (36, 37) and size (36).

Integrating QTT analysis and DEGs phenotypes in knockout mice using the Mouse Genome Informatics database, three genes were identified as candidate genes in the pituitary gland that may influence the chicken growth and carcass traits. For example, The knockout mice were smaller than wild-type littermates during the first postnatal weeks, suggesting that knocking out the *GRIA1* gene might result in decreased body size (38). *IRF4* has been reported to be associated with growth in relation to increasing body weight (39). And it also plays a wide variety of roles in the immune system, including regulation of T cell function (40, 41) and differentiation of CD4+ and CD8+ T cells (42–44). As we know, growth and the immune system are complex and essential for the lives of animals, and more research is needed on the balance between growth and immunity.

*PRKG2* (cGMP-dependent protein kinase type II) is a key regulatory kinase in temporal and spatial development of growth plate cartilage (45). Loss of *PRKG2* function results in decreased body weight and body length (46). Additionally, the functional role of *PRKG2* in growth plate development is highly conserved across many species. For example, knockout mice and naturally occurring mutations in *PRKG2* in rats and cattle exhibited achondroplastic dwarfism (46–48). A deletion of *PRKG2* on human chromosome 4q21 was demonstrated to be associated with growth restriction (49). However, the function of *PRKG2* (especially in Chinese local chickens) remains unclear. RNA-seq analysis and the results of qRT–PCR in this study showed that *PRKG2* was significantly differentially expressed between the H and L groups. The QTT analysis also identified *PRKG2* as an important candidate gene. Overall, these results suggest that *PRKG2* might play a crucial role in chicken growth. Furthermore, it was observed that the SNP *rs16400745* mutation (TT) had positive effects on almost all growth and carcass traits in the Ningdu yellow chicken population. At the same time, Ningdu yellow chickens carry the mutation TT at relatively low frequencies (0.10). Ultimately, this study provides a theoretical reference for the molecular-assisted breeding of desirable chickens.

## Conclusion

In this study, 399 Chinese Ningdu yellow chickens were phenotyped and high correlations of growth and carcass traits were found. A total of 428 genes were identified to be differentially expressed between the H and L groups through RNA-seq analysis. A functional analysis of the DEGs identified a significant enrichment of 158 GO terms (i.e., response to stimulus) and two KEGG pathways (i.e., neuroactive ligand-receptor interaction). The qRT-PCR, QTT and association analyses identified *PRKG2* as a candidate gene underlying growth and carcass traits in Chinese Ningdu yellow chickens. These novel findings provide the first insight into the genetic basis of growth in indigenous Chinese Ningdu yellow chickens and are likely to serve as a theoretical reference for the molecular-assisted breeding of desirable chickens.

## Data Availability Statement

The original contributions presented in the study are publicly available. This data can be found here: SRA accession: PRJNA814327.

## Ethics Statement

The animal study was reviewed and approved by Animal Care Committee of Nanchang Normal University.

## Author Contributions

XX conceived and designed the experiments, analyzed the data, and wrote and revised the manuscript. MZ, XZ, and YT performed the experiments. ZW analyzed the data. JG and JX collected the samples. YW and JL performed the experiments. XT revised the manuscript. YR conceived and designed the experiments and revised the manuscript. All authors contributed to the article and approved the submitted version.

## Funding

This work was supported by the National Natural Science Foundation of China (32060740), the Key Research and Development Program of Jiangxi Province (20212BBF63028), and the Doctoral Foundation of Nanchang Normal University (NSBSJJ2019009).

## Conflict of Interest

The authors declare that the research was conducted in the absence of any commercial or financial relationships that could be construed as a potential conflict of interest.

## Publisher's Note

All claims expressed in this article are solely those of the authors and do not necessarily represent those of their affiliated organizations, or those of the publisher, the editors and the reviewers. Any product that may be evaluated in this article, or claim that may be made by its manufacturer, is not guaranteed or endorsed by the publisher.
