# Supplementary material for: RNA Sequencing of the Pituitary Gland and Association Analyses Reveal PRKG2 as a Candidate Gene for Growth and Carcass Traits in Chinese Ningdu Yellow Chickens
Source: Front Vet Sci. 2022 Jun 16;9:892024. doi: 10.3389/fvets.2022.892024 (PMC9244401; doi:10.3389/fvets.2022.892024)
Supplement: Supplementary file 1 [file Table_1.DOCX]

**Supplementary Table S1. The phenotypic data for the 12 chickens.**

| **Groups** | **Sample name** | **Live weight (g)** | **Carcass weight (g)** |
| --- | --- | --- | --- |
| **H** | N_T01 | 1275.3 | 1139.3 |
|  | N_T02 | 1234.6 | 1057.1 |
|  | N_T03 | 1197.4 | 961.7 |
|  | N_T04 | 1171.1 | 1011.0 |
|  | N_T05 | 1159.3 | 1038.0 |
|  | N_T06 | 1168.0 | 954.7 |
| **L** | N_T07 | 566.4 | 496.9 |
|  | N_T08 | 602.3 | 529.5 |
|  | N_T09 | 613.3 | 529.4 |
|  | N_T10 | 620.3 | 559.0 |
|  | N_T11 | 647.7 | 549.0 |
|  | N_T12 | 649.6 | 544.3 |

**Supplementary Table S2. Primers used in qRT-PCR and PCR amplification of chicken genes.**

|  | **Gene Name** | **(5'-3') Sequence** | **Product size (bp)** |
| --- | --- | --- | --- |
| **qRT-PCR** | *GH* | FP: CCTACCTGAAGGTGATGAAGTGCCG  RP: CTGTGGGGTTTATTCCTCGTGTTTT | 147 |
|  | *IRF4* | FP: CCCAAACTAAGACAGCCAAGGA  RP: CCACACAGACTCAACAGACCCC | 144 |
|  | *NPBWR2* | FP: GTCTGTGCCAACTTCTGCGTGC  RP: TGCTTCTAATCAGTGCCTTTCT | 158 |
|  | *NRROS* | FP: TTGAACCCAACCACCACAAGCA  RP: GCGGAAGGAGCAAACGGAAACT | 184 |
|  | *PRKG2* | FP: GAACCCAACAGAAAGATTAGGCA  RP: TAAGCAAGAAACACAAGAACAGG | 231 |
|  | *ENSGALG00000051068* | FP: CCCCTCCTCACCGCCTTCC  RP: TCCCCCAGCGTCACCTCCC | 215 |
|  | *GAPDH* | CGCCATCACTATCTTCCAGGAGC  CGCTTAGCACCACCCTTCAGATG | 138 |
| **PCR** | *PRKG2* | FP: TAAAGACTCCGAAACTCACT  RP: ACGCACCATAGACTCATT | 950 |

**Supplementary Table S3. The correlation values among 17 traits related to growth and carcass through correlation analysis.**

|  | Birth weight | Live weight | Carcass weight | Semi-eviscerated weight | Eviscerated weight | Breast muscle weight | Carcass rate | Semi-eviscerated rate | Eviscerated rate | Breast muscle rate | Average daily gain | Body length | Chest circumference | Chest depth | Chest angle | Chest width | Back width |
| --- | --- | --- | --- | --- | --- | --- | --- | --- | --- | --- | --- | --- | --- | --- | --- | --- | --- |
| Birth weight | 1.00 | 0.17 | 0.11 | 0.10 | 0.09 | 0.11 | -0.04 | -0.05 | -0.05 | 0.03 | 0.14 | 0.04 | 0.00 | 0.03 | 0.12 | 0.01 | -0.04 |
| Live weight | 0.17 | 1.00 | 0.77 | 0.76 | 0.77 | 0.64 | -0.11 | -0.08 | 0.00 | 0.16 | 1.00 | 0.56 | 0.47 | 0.39 | 0.20 | 0.17 | 0.36 |
| Carcass weight | 0.11 | 0.77 | 1.00 | 0.95 | 0.95 | 0.80 | 0.55 | 0.50 | 0.55 | 0.50 | 0.77 | 0.68 | 0.61 | 0.51 | 0.27 | 0.17 | 0.40 |
| Semi-eviscerated weight | 0.10 | 0.76 | 0.95 | 1.00 | 0.98 | 0.83 | 0.48 | 0.58 | 0.61 | 0.54 | 0.76 | 0.69 | 0.64 | 0.48 | 0.27 | 0.16 | 0.43 |
| Eviscerated weight | 0.09 | 0.77 | 0.95 | 0.98 | 1.00 | 0.87 | 0.46 | 0.54 | 0.63 | 0.59 | 0.76 | 0.71 | 0.67 | 0.48 | 0.28 | 0.17 | 0.45 |
| Breast muscle weight | 0.11 | 0.64 | 0.80 | 0.83 | 0.87 | 1.00 | 0.39 | 0.48 | 0.58 | 0.85 | 0.62 | 0.59 | 0.64 | 0.38 | 0.26 | 0.19 | 0.34 |
| Carcass rate | -0.04 | -0.11 | 0.55 | 0.48 | 0.46 | 0.39 | 1.00 | 0.88 | 0.85 | 0.56 | -0.15 | 0.33 | 0.33 | 0.28 | 0.16 | 0.04 | 0.15 |
| Semi-eviscerated rate | -0.05 | -0.08 | 0.50 | 0.58 | 0.54 | 0.48 | 0.88 | 1.00 | 0.95 | 0.65 | -0.13 | 0.36 | 0.39 | 0.25 | 0.16 | 0.03 | 0.22 |
| Eviscerated rate | -0.05 | 0.00 | 0.55 | 0.61 | 0.63 | 0.58 | 0.85 | 0.95 | 1.00 | 0.74 | -0.05 | 0.43 | 0.48 | 0.28 | 0.18 | 0.05 | 0.26 |
| Breast muscle rate | 0.03 | 0.16 | 0.50 | 0.54 | 0.59 | 0.85 | 0.56 | 0.65 | 0.74 | 1.00 | 0.12 | 0.38 | 0.50 | 0.23 | 0.19 | 0.13 | 0.20 |
| Average daily gain | 0.14 | 1.00 | 0.77 | 0.76 | 0.76 | 0.62 | -0.15 | -0.13 | -0.05 | 0.12 | 1.00 | 0.53 | 0.44 | 0.41 | 0.18 | 0.19 | 0.38 |
| Body length | 0.04 | 0.56 | 0.68 | 0.69 | 0.71 | 0.59 | 0.33 | 0.36 | 0.43 | 0.38 | 0.53 | 1.00 | 0.47 | 0.41 | 0.13 | 0.20 | 0.39 |
| Chest circumference | 0.00 | 0.47 | 0.61 | 0.64 | 0.67 | 0.64 | 0.33 | 0.39 | 0.48 | 0.50 | 0.44 | 0.47 | 1.00 | 0.24 | 0.14 | 0.43 | 0.45 |
| Chest depth | 0.03 | 0.39 | 0.51 | 0.48 | 0.48 | 0.38 | 0.28 | 0.25 | 0.28 | 0.23 | 0.41 | 0.41 | 0.24 | 1.00 | 0.13 | 0.07 | 0.09 |
| Chest angle | 0.12 | 0.20 | 0.27 | 0.27 | 0.28 | 0.26 | 0.16 | 0.16 | 0.18 | 0.19 | 0.18 | 0.13 | 0.14 | 0.13 | 1.00 | -0.18 | -0.16 |
| Chest width | 0.01 | 0.17 | 0.17 | 0.16 | 0.17 | 0.19 | 0.04 | 0.03 | 0.05 | 0.13 | 0.19 | 0.20 | 0.43 | 0.07 | -0.18 | 1.00 | 0.39 |
| Back width | -0.04 | 0.36 | 0.40 | 0.43 | 0.45 | 0.34 | 0.15 | 0.22 | 0.26 | 0.20 | 0.38 | 0.39 | 0.45 | 0.09 | -0.16 | 0.39 | 1.00 |

**Supplementary Table S4. The *P* values among 17 traits related to growth and carcass through correlation analysis.**

|  | Birth weight | Live weight | Carcass weight | Semi-eviscerated weight | Eviscerated weight | Breast muscle weight | Carcass rate | Semi-eviscerated rate | Eviscerated rate | Breast muscle rate | Average daily gain | Body length | Chest circumference | Chest depth | Chest angle | Chest width | Back width |
| --- | --- | --- | --- | --- | --- | --- | --- | --- | --- | --- | --- | --- | --- | --- | --- | --- | --- |
| Birth weight | 0.00E+00 | 1.76E-03 | 4.95E-02 | 5.74E-02 | 8.27E-02 | 4.81E-02 | 4.13E-01 | 3.43E-01 | 4.06E-01 | 5.67E-01 | 1.08E-02 | 4.11E-01 | 9.62E-01 | 5.89E-01 | 2.72E-02 | 8.75E-01 | 4.32E-01 |
| Live weight | 1.76E-03 | 0.00E+00 | 7.12E-78 | 1.07E-76 | 3.09E-78 | 4.71E-46 | 2.46E-02 | 1.21E-01 | 9.82E-01 | 1.80E-03 | 0.00E+00 | 3.44E-34 | 1.41E-23 | 6.31E-16 | 5.42E-05 | 5.95E-04 | 8.28E-14 |
| Carcass weight | 4.95E-02 | 7.12E-78 | 0.00E+00 | 4.12E-202 | 1.84E-192 | 1.28E-86 | 1.77E-32 | 1.23E-26 | 1.72E-32 | 6.25E-26 | 5.21E-67 | 7.26E-56 | 1.82E-42 | 1.11E-27 | 3.93E-08 | 6.19E-04 | 8.13E-17 |
| Semi-eviscerated weight | 5.74E-02 | 1.07E-76 | 4.12E-202 | 0.00E+00 | 9.36E-276 | 5.75E-101 | 9.55E-24 | 1.70E-36 | 1.14E-40 | 4.11E-31 | 5.00E-65 | 1.60E-57 | 1.64E-46 | 1.57E-24 | 5.44E-08 | 1.11E-03 | 3.42E-19 |
| Eviscerated weight | 8.27E-02 | 3.09E-78 | 1.84E-192 | 9.36E-276 | 0.00E+00 | 1.73E-118 | 6.46E-22 | 1.94E-31 | 1.77E-45 | 1.91E-37 | 2.14E-64 | 5.37E-62 | 9.20E-53 | 5.97E-24 | 2.20E-08 | 6.29E-04 | 7.51E-21 |
| Breast muscle weight | 4.81E-02 | 4.71E-46 | 1.28E-86 | 5.75E-101 | 1.73E-118 | 0.00E+00 | 8.86E-16 | 1.02E-23 | 8.03E-37 | 1.40E-112 | 2.68E-36 | 4.28E-38 | 1.98E-46 | 8.06E-15 | 1.35E-07 | 1.13E-04 | 2.50E-12 |
| Carcass rate | 4.13E-01 | 2.46E-02 | 1.77E-32 | 9.55E-24 | 6.46E-22 | 8.86E-16 | 0.00E+00 | 3.07E-130 | 7.75E-113 | 6.50E-34 | 5.26E-03 | 2.98E-11 | 7.97E-12 | 1.39E-08 | 1.43E-03 | 4.60E-01 | 3.40E-03 |
| Semi-eviscerated rate | 3.43E-01 | 1.21E-01 | 1.23E-26 | 1.70E-36 | 1.94E-31 | 1.02E-23 | 3.07E-130 | 0.00E+00 | 1.80E-193 | 1.34E-48 | 2.02E-02 | 2.26E-13 | 4.76E-16 | 2.91E-07 | 1.03E-03 | 5.08E-01 | 1.56E-05 |
| Eviscerated rate | 4.06E-01 | 9.82E-01 | 1.72E-32 | 1.14E-40 | 1.77E-45 | 8.03E-37 | 7.75E-113 | 1.80E-193 | 0.00E+00 | 5.31E-68 | 3.80E-01 | 1.92E-19 | 4.73E-24 | 2.86E-08 | 2.57E-04 | 2.80E-01 | 1.39E-07 |
| Breast muscle rate | 5.67E-01 | 1.80E-03 | 6.25E-26 | 4.11E-31 | 1.91E-37 | 1.40E-112 | 6.50E-34 | 1.34E-48 | 5.31E-68 | 0.00E+00 | 2.99E-02 | 1.13E-14 | 3.01E-26 | 7.00E-06 | 1.05E-04 | 8.91E-03 | 4.86E-05 |
| Average daily gain | 1.08E-02 | 0.00E+00 | 5.21E-67 | 5.00E-65 | 2.14E-64 | 2.68E-36 | 5.26E-03 | 2.02E-02 | 3.80E-01 | 2.99E-02 | 0.00E+00 | 1.67E-26 | 6.43E-18 | 2.57E-15 | 9.98E-04 | 3.75E-04 | 4.91E-13 |
| Body length | 4.11E-01 | 3.44E-34 | 7.26E-56 | 1.60E-57 | 5.37E-62 | 4.28E-38 | 2.98E-11 | 2.26E-13 | 1.92E-19 | 1.13E-14 | 1.67E-26 | 0.00E+00 | 5.94E-23 | 6.42E-18 | 1.15E-02 | 4.78E-05 | 4.96E-16 |
| Chest circumference | 9.62E-01 | 1.41E-23 | 1.82E-42 | 1.64E-46 | 9.20E-53 | 1.98E-46 | 7.97E-12 | 4.76E-16 | 4.73E-24 | 3.01E-26 | 6.43E-18 | 5.94E-23 | 0.00E+00 | 1.62E-06 | 5.83E-03 | 1.01E-19 | 6.01E-21 |
| Chest depth | 5.89E-01 | 6.31E-16 | 1.11E-27 | 1.57E-24 | 5.97E-24 | 8.06E-15 | 1.39E-08 | 2.91E-07 | 2.86E-08 | 7.00E-06 | 2.57E-15 | 6.42E-18 | 1.62E-06 | 0.00E+00 | 9.60E-03 | 1.89E-01 | 7.75E-02 |
| Chest angle | 2.72E-02 | 5.42E-05 | 3.93E-08 | 5.44E-08 | 2.20E-08 | 1.35E-07 | 1.43E-03 | 1.03E-03 | 2.57E-04 | 1.05E-04 | 9.98E-04 | 1.15E-02 | 5.83E-03 | 9.60E-03 | 0.00E+00 | 4.27E-04 | 1.13E-03 |
| Chest width | 8.75E-01 | 5.95E-04 | 6.19E-04 | 1.11E-03 | 6.29E-04 | 1.13E-04 | 4.60E-01 | 5.08E-01 | 2.80E-01 | 8.91E-03 | 3.75E-04 | 4.78E-05 | 1.01E-19 | 1.89E-01 | 4.27E-04 | 0.00E+00 | 2.74E-16 |
| Back width | 4.32E-01 | 8.28E-14 | 8.13E-17 | 3.42E-19 | 7.51E-21 | 2.50E-12 | 3.40E-03 | 1.56E-05 | 1.39E-07 | 4.86E-05 | 4.91E-13 | 4.96E-16 | 6.01E-21 | 7.75E-02 | 1.13E-03 | 2.74E-16 | 0.00E+00 |

**Supplementary Table S5.** Sequence quality and alignment information of 12 pituitary gland samples in the H group and the L group

| **Sample name** | **Raw reads** | **Clean reads** | **Clean bases (G)** | **GC content (%)** | **Total mapped (Percentage)** | **Multiple mapped (Percentage)** |
| --- | --- | --- | --- | --- | --- | --- |
| N_T01 | 43,577,762 | 42,038,094 | 6.31 | 52.61 | 35,981,501 (85.59%) | 712,307 (1.69%) |
| N_T02 | 55,695,900 | 54,307,040 | 8.15 | 51.59 | 49,177,413 (90.55%) | 902,828 (1.66%) |
| N_T03 | 45,214,460 | 43,979,020 | 6.60 | 50.86 | 39,087,640 (88.88%) | 746,678 (1.70%) |
| N_T04 | 42,991,088 | 42,653,880 | 6.40 | 51.10 | 38,684,949 (90.70%) | 611,148 (1.43%) |
| N_T05 | 45,502,118 | 44,287,482 | 6.64 | 50.33 | 40,273,235 (90.94%) | 713,418 (1.61%) |
| N_T06 | 46,484,824 | 46,101,450 | 6.92 | 51.53 | 41,239,908 (89.45%) | 728,937 (1.58%) |
| N_T07 | 39,744,928 | 39,239,334 | 5.89 | 50.58 | 35,619,409 (90.77%) | 531,322 (1.35%) |
| N_T08 | 48,264,740 | 46,863,404 | 7.03 | 50.36 | 42,069,767 (89.77%) | 734,871 (1.57%) |
| N_T09 | 49,296,776 | 47,797,586 | 7.17 | 50.77 | 42,573,677 (89.07%) | 750,388 (1.57%) |
| N_T10 | 50,531,972 | 49,056,426 | 7.36 | 51.11 | 43,963,533 (89.62%) | 840,522 (1.71%) |
| N_T11 | 42,399,864 | 41,121,640 | 6.17 | 50.64 | 36,964,272 (89.89%) | 633,476 (1.54%) |
| N_T12 | 46,925,090 | 46,420,640 | 6.96 | 50.84 | 40,986,522 (88.29%) | 813,790 (1.75%) |

**Supplementary Table S6. The differentially expressed genes (DEGs) between the H and L groups.**

| **Gene id** | **Gene name** | **Gene locus** | **log2FoldChange** | **FDR** |
| --- | --- | --- | --- | --- |
| ENSGALG00000036395 | IGHVL | 27:2272709-2649530 | -1.62 | 5.62E-07 |
| ENSGALG00000030546 | CLEC19A | 14:8815486-8821411 | -2.19 | 7.85E-07 |
| ENSGALG00000014754 | ENSGALG00000014754 | 1:78777330-78971694 | -1.80 | 1.12E-06 |
| ENSGALG00000033932 | BF1 | 16:2565405-2595262 | -1.42 | 1.86E-06 |
| ENSGALG00000010269 | KCNAB1 | 9:23040155-23068606 | -1.09 | 2.53E-06 |
| ENSGALG00000014908 | FST | Z:16072541-16079179 | -3.70 | 2.97E-06 |
| ENSGALG00000012830 | IRF4 | 2:66457756-66470687 | -3.40 | 5.88E-06 |
| ENSGALG00000031652 | AK5 | 8:19432096-19518959 | -1.72 | 8.39E-06 |
| ENSGALG00000049450 | ENSGALG00000049450 | 15:8214032-8214343 | -6.01 | 8.68E-06 |
| ENSGALG00000039080 | CD44 | 5:19191268-19248208 | -1.45 | 9.02E-06 |
| ENSGALG00000050515 | ENSGALG00000050515 | KZ626833.1:112024-112371 | -6.55 | 1.00E-05 |
| ENSGALG00000012456 | RAC2 | 1:51413962-51423737 | -1.71 | 1.35E-05 |
| ENSGALG00000011551 | JCHAIN | 4:50012279-50018146 | -5.21 | 1.44E-05 |
| ENSGALG00000041611 | ENSGALG00000041611 | 23:313700-315602 | -1.75 | 1.50E-05 |
| ENSGALG00000047395 | ENSGALG00000047395 | 15:8217930-8218211 | -5.21 | 1.82E-05 |
| ENSGALG00000016986 | LCP1 | 1:169350498-169399736 | -1.38 | 2.18E-05 |
| ENSGALG00000051068 | ENSGALG00000051068 | 4:91264001-91278824 | -1.65 | 2.31E-05 |
| ENSGALG00000030571 | ENSGALG00000030571 | 1:168025737-168042111 | -1.68 | 2.74E-05 |
| ENSGALG00000025945 | AVD | Z:9164659-9166088 | -3.28 | 2.79E-05 |
| ENSGALG00000004167 | ENSGALG00000004167 | 6:11522560-11524423 | -2.01 | 2.79E-05 |
| ENSGALG00000030940 | BLB2 | 16:2572332-2573868 | -1.51 | 5.67E-05 |
| ENSGALG00000039682 | HCLS1 | 1:359020-371025 | -1.44 | 6.52E-05 |
| ENSGALG00000001373 | TRAF3IP3 | 26:3154638-3288987 | -1.14 | 6.98E-05 |
| ENSGALG00000014603 | C1S | 1:78045961-78055022 | -1.19 | 7.10E-05 |
| ENSGALG00000050240 | TBX21 | 27:6538026-6545277 | -1.95 | 7.14E-05 |
| ENSGALG00000049716 | ENSGALG00000049716 | 31:2336416-2336720 | -4.44 | 7.20E-05 |
| ENSGALG00000012791 | TBXAS1 | 1:55750244-55990181 | -1.38 | 8.20E-05 |
| ENSGALG00000000141 | BLB1 | 16:2565094-2566926 | -1.47 | 9.59E-05 |
| ENSGALG00000006583 | LSP1 | 5:14250735-14289860 | -1.30 | 1.03E-04 |
| ENSGALG00000008447 | SH2D1A | 4:15530219-15544105 | -1.85 | 1.14E-04 |
| ENSGALG00000051946 | ENSGALG00000051946 | 31:2331784-2332086 | -4.57 | 1.21E-04 |
| ENSGALG00000004594 | Ii | 13:13851648-13855558 | -1.23 | 1.21E-04 |
| ENSGALG00000037856 | CHL1 | 12:17603136-17707996 | -1.09 | 1.27E-04 |
| ENSGALG00000023435 | GATM | 10:11349046-11362107 | -1.78 | 1.38E-04 |
| ENSGALG00000001571 | MYO1F | 28:1844413-1858231 | -1.37 | 1.73E-04 |
| ENSGALG00000032469 | ARHGAP45 | 28:3019600-3031885 | -1.11 | 1.73E-04 |
| ENSGALG00000052196 | ENSGALG00000052196 | 23:6036756-6053909 | -1.27 | 1.84E-04 |
| ENSGALG00000050545 | ENSGALG00000050545 | 15:8226219-8226506 | -5.80 | 1.90E-04 |
| ENSGALG00000048390 | ENSGALG00000048390 | 15:8229080-8229361 | -5.23 | 2.04E-04 |
| ENSGALG00000013548 | GZMA | Z:16643589-16666277 | -2.76 | 2.06E-04 |
| ENSGALG00000049890 | ENSGALG00000049890 | 2:104569564-104719148 | -1.45 | 2.22E-04 |
| ENSGALG00000012056 | GRAP2 | 1:50298523-50339266 | -1.77 | 2.92E-04 |
| ENSGALG00000025886 | SUSD3 | 12:7057412-7082243 | -1.41 | 2.99E-04 |
| ENSGALG00000052514 | ENSGALG00000052514 | 28:3535041-3536298 | -1.51 | 3.08E-04 |
| ENSGALG00000007511 | ITGB2 | 7:7177686-7189495 | -1.15 | 3.28E-04 |
| ENSGALG00000002192 | PTPRC | 8:2418766-2492350 | -1.09 | 3.45E-04 |
| ENSGALG00000006809 | POU2AF1 | 24:4343410-4355489 | -3.04 | 3.50E-04 |
| ENSGALG00000007418 | CD3D | 24:5487325-5489870 | -1.64 | 3.67E-04 |
| ENSGALG00000005638 | IL2RG | 4:2390550-2394261 | -1.84 | 3.84E-04 |
| ENSGALG00000026152 | ENSGALG00000026152 | 12:2699650-2704153 | -1.34 | 4.13E-04 |
| ENSGALG00000007780 | EIF4E3 | 12:16666749-16682429 | -1.04 | 4.18E-04 |
| ENSGALG00000002080 | DOCK2 | 13:4712213-4872139 | -1.06 | 4.34E-04 |
| ENSGALG00000001486 | ZAP70 | 28:1911962-1933263 | -1.55 | 4.46E-04 |
| ENSGALG00000033694 | IFI30 | 28:4098288-4100719 | -1.53 | 4.65E-04 |
| ENSGALG00000013575 | IFI6 | 2:89717661-89722211 | -1.48 | 4.81E-04 |
| ENSGALG00000016964 | ENSGALG00000016964 | 1:167989491-168020290 | -1.40 | 5.33E-04 |
| ENSGALG00000047227 | ENSGALG00000047227 | 31:2361772-2362074 | -5.83 | 5.52E-04 |
| ENSGALG00000054971 | ARHGAP30 | 25:20565-29413 | -1.68 | 5.67E-04 |
| ENSGALG00000049444 | ENSGALG00000049444 | 16:2069172-2071163 | -3.19 | 6.19E-04 |
| ENSGALG00000007543 | APCDD1L | 20:11158900-11172710 | -1.74 | 6.22E-04 |
| ENSGALG00000007416 | CD3E | 24:5480338-5485277 | -1.41 | 6.47E-04 |
| ENSGALG00000006453 | TF | 9:4765436-4777129 | -1.34 | 6.74E-04 |
| ENSGALG00000037929 | BIN2 | 33:5033336-5044090 | -1.67 | 7.48E-04 |
| ENSGALG00000005644 | MYO1G | 2:4129233-4138053 | -1.48 | 7.66E-04 |
| ENSGALG00000030602 | ADAM33 | 4:89568221-89591688 | -1.11 | 7.85E-04 |
| ENSGALG00000014585 | ENSGALG00000014585 | 19:4941785-4942879 | -2.51 | 8.54E-04 |
| ENSGALG00000035075 | TAP1 | 16:2596216-2601012 | -1.01 | 8.59E-04 |
| ENSGALG00000006756 | ENSGALG00000006756 | 20:10341499-10348637 | -1.24 | 9.05E-04 |
| ENSGALG00000002113 | LCP2 | 13:4598169-4629140 | -1.21 | 9.06E-04 |
| ENSGALG00000050376 | ENSGALG00000050376 | 15:8226765-8227052 | -5.64 | 9.14E-04 |
| ENSGALG00000028256 | CCL19 | Z:8363698-8366694 | -2.10 | 1.02E-03 |
| ENSGALG00000046192 | CCL19 | Z:8370305-8373001 | -1.52 | 1.04E-03 |
| ENSGALG00000004290 | ANGPTL1 | 8:6716174-6733176 | -1.03 | 1.06E-03 |
| ENSGALG00000013956 | MYB | 3:55778483-55804513 | -3.43 | 1.11E-03 |
| ENSGALG00000027165 | RNASE4 | 6:9958422-9959631 | -1.64 | 1.19E-03 |
| ENSGALG00000051101 | ENSGALG00000051101 | 23:5959344-6092916 | -1.24 | 1.24E-03 |
| ENSGALG00000033278 | NLRC3 | 14:13150266-13163527 | -1.54 | 1.25E-03 |
| ENSGALG00000044326 | ENSGALG00000044326 | 31:4845515-4866801 | -1.58 | 1.29E-03 |
| ENSGALG00000013546 | ENSGALG00000013546 | Z:16648999-16663409 | -1.81 | 1.30E-03 |
| ENSGALG00000035386 | NCKAP1L | 33:6036926-6046319 | -1.40 | 1.30E-03 |
| ENSGALG00000053057 | ENSGALG00000053057 | 15:8230684-8230971 | -5.95 | 1.31E-03 |
| ENSGALG00000031916 | ZP1 | 5:264555-268945 | -1.26 | 1.32E-03 |
| ENSGALG00000034505 | CDKN2A | Z:78846780-78856728 | -2.20 | 1.37E-03 |
| ENSGALG00000016142 | MX1 | 1:110239567-110260524 | -1.30 | 1.38E-03 |
| ENSGALG00000043016 | ENSGALG00000043016 | 5:283574-288430 | -2.26 | 1.40E-03 |
| ENSGALG00000049938 | ENSGALG00000049938 | 31:2377704-2378006 | -5.83 | 1.41E-03 |
| ENSGALG00000041094 | SYT1 | 1:39190683-39534902 | -1.22 | 1.51E-03 |
| ENSGALG00000013723 | OASL | 12:3635427-3637460 | -1.72 | 1.55E-03 |
| ENSGALG00000023689 | ASS1 | 17:6180976-6201571 | -1.08 | 1.69E-03 |
| ENSGALG00000020388 | ENSGALG00000020388 | 5:45800125-45803318 | -2.19 | 1.81E-03 |
| ENSGALG00000031359 | GCK | 22:5253936-5261455 | -1.13 | 1.83E-03 |
| ENSGALG00000036093 | PTPN7 | 26:367531-376406 | -1.47 | 1.83E-03 |
| ENSGALG00000028448 | SH2D1B | 1:88856191-88866344 | -1.49 | 1.90E-03 |
| ENSGALG00000047283 | ENSGALG00000047283 | 31:4827950-4839801 | -1.81 | 1.97E-03 |
| ENSGALG00000042227 | GNLY | 22:5262428-5263904 | -1.13 | 2.00E-03 |
| ENSGALG00000020331 | ENSGALG00000020331 | 4:1435125-1442502 | -1.79 | 2.02E-03 |
| ENSGALG00000012545 | CYTIP | 7:36217989-36226734 | -1.30 | 2.02E-03 |
| ENSGALG00000050564 | ENSGALG00000050564 | 6:19358682-19386012 | -1.13 | 2.07E-03 |
| ENSGALG00000045085 | IFIT5 | 6:20187816-20191669 | -1.50 | 2.09E-03 |
| ENSGALG00000005209 | AQP1 | 2:1186169-1206259 | -1.48 | 2.11E-03 |
| ENSGALG00000000249 | GH | 27:4034905-4038410 | -2.33 | 2.19E-03 |
| ENSGALG00000005257 | ENSGALG00000005257 | 8:12271795-12279181 | -1.05 | 2.21E-03 |
| ENSGALG00000051167 | ENSGALG00000051167 | 15:8223554-8223835 | -4.76 | 2.22E-03 |
| ENSGALG00000008638 | MMR1L3 | 2:19497094-19526728 | -1.48 | 2.23E-03 |
| ENSGALG00000007186 | TMEM268 | 17:2882867-2987652 | -1.20 | 2.27E-03 |
| ENSGALG00000038393 | DMB2 | 16:2589290-2592336 | -1.13 | 2.29E-03 |
| ENSGALG00000027407 | ENSGALG00000027407 | 5:52154934-52170189 | -2.13 | 2.36E-03 |
| ENSGALG00000032746 | ENPP2 | 2:136526177-136595461 | -1.05 | 2.42E-03 |
| ENSGALG00000039156 | ENSGALG00000039156 | 30:770562-784767 | -1.56 | 2.42E-03 |
| ENSGALG00000054486 | MC1R | 11:18840387-18841759 | -4.53 | 2.43E-03 |
| ENSGALG00000005094 | GRAP | 14:5564574-5568596 | -1.50 | 2.46E-03 |
| ENSGALG00000012119 | MARCO | 7:28482051-28491954 | -1.74 | 2.50E-03 |
| ENSGALG00000048325 | GBP | 12:2706975-2709494 | -1.89 | 2.59E-03 |
| ENSGALG00000049747 | ENSGALG00000049747 | 31:2385270-2385572 | -6.40 | 2.60E-03 |
| ENSGALG00000034085 | ENSGALG00000034085 | 20:1160252-1181245 | -1.76 | 2.62E-03 |
| ENSGALG00000009639 | DDX60 | 4:24959132-24997825 | -1.54 | 2.64E-03 |
| ENSGALG00000053601 | LPXN | 5:17046436-17193688 | -1.01 | 2.74E-03 |
| ENSGALG00000001181 | KCNJ5 | 24:1097586-1142577 | -1.15 | 2.77E-03 |
| ENSGALG00000016721 | PLCXD1 | 1:131370527-131395496 | -1.23 | 2.83E-03 |
| ENSGALG00000035856 | CD48 | 25:2468024-2470555 | -1.36 | 2.87E-03 |
| ENSGALG00000016022 | RUNX1 | 1:107358560-107506805 | -1.02 | 2.99E-03 |
| ENSGALG00000006229 | ENSGALG00000006229 | 25:2452489-2458591 | -1.32 | 2.99E-03 |
| ENSGALG00000004859 | ZNFX1 | 20:6715285-6727531 | -1.03 | 3.03E-03 |
| ENSGALG00000033234 | JAK3 | 28:3422187-3433690 | -1.55 | 3.12E-03 |
| ENSGALG00000006325 | ENSGALG00000006325 | 12:11116418-11157789 | -1.55 | 3.32E-03 |
| ENSGALG00000054189 | ENSGALG00000054189 | 31:2409395-2409694 | -6.47 | 3.39E-03 |
| ENSGALG00000043172 | CLVS1 | 2:113184668-113281852 | -1.53 | 3.45E-03 |
| ENSGALG00000010598 | KCNK10 | 5:43170624-43224081 | -1.03 | 3.56E-03 |
| ENSGALG00000052643 | C15orf48 | 10:11117010-11120725 | -1.31 | 3.63E-03 |
| ENSGALG00000033087 | STAT4 | 7:7937367-7974968 | -1.22 | 3.64E-03 |
| ENSGALG00000021627 | IFI27L1 | 23:4490448-4491440 | -1.31 | 3.65E-03 |
| ENSGALG00000021569 | C1QA | 21:6079357-6081398 | -1.52 | 3.66E-03 |
| ENSGALG00000030432 | RASSF2 | 22:559504-571288 | -1.05 | 3.66E-03 |
| ENSGALG00000032701 | LY96 | 2:118035819-118043555 | -1.60 | 3.70E-03 |
| ENSGALG00000010672 | KCNK13 | 5:43917283-43969356 | -1.29 | 3.73E-03 |
| ENSGALG00000010595 | GPR65 | 5:43100745-43106712 | -1.55 | 3.76E-03 |
| ENSGALG00000015712 | SLBP | 4:83930355-83937106 | -1.67 | 3.77E-03 |
| ENSGALG00000043372 | RUNX3 | 23:2591781-2635428 | -1.30 | 3.81E-03 |
| ENSGALG00000028496 | ENSGALG00000028496 | 24:4335489-4342070 | -4.21 | 3.85E-03 |
| ENSGALG00000047211 | FCER1G | 25:152758-153848 | -1.66 | 4.06E-03 |
| ENSGALG00000032428 | ENSGALG00000032428 | 11:8389347-8396105 | -1.25 | 4.08E-03 |
| ENSGALG00000014372 | SLC34A2 | 4:73421043-73440963 | -1.91 | 4.11E-03 |
| ENSGALG00000008127 | SPI1 | 5:23185094-23206765 | -1.09 | 4.17E-03 |
| ENSGALG00000048032 | ENSGALG00000048032 | 2:28587291-28590156 | -1.11 | 4.52E-03 |
| ENSGALG00000052142 | ENSGALG00000052142 | 31:2369628-2369930 | -5.04 | 4.93E-03 |
| ENSGALG00000003876 | TIMD4 | 13:12172827-12188986 | -1.49 | 4.94E-03 |
| ENSGALG00000024122 | TNFRSF13B | 14:5052186-5055318 | -3.01 | 5.02E-03 |
| ENSGALG00000050893 | CCR2 | 2:42632317-42640047 | -1.29 | 5.10E-03 |
| ENSGALG00000027247 | EOMES | 2:38541856-38546582 | -2.07 | 5.26E-03 |
| ENSGALG00000002329 | CCL1 | 19:4939772-4941235 | -1.98 | 5.59E-03 |
| ENSGALG00000026884 | ENSGALG00000026884 | Z:7161279-7165204 | -1.91 | 5.73E-03 |
| ENSGALG00000004958 | BTK | 4:2028580-2039548 | -1.16 | 5.73E-03 |
| ENSGALG00000026995 | RHOG | 1:197093201-197097605 | -1.35 | 5.76E-03 |
| ENSGALG00000028659 | BLEC1 | 16:2562353-2565190 | -1.82 | 5.78E-03 |
| ENSGALG00000013409 | ENSGALG00000013409 | 23:2220746-2229123 | -1.43 | 5.88E-03 |
| ENSGALG00000027747 | TROJANZ | Z:9025003-9030333 | -1.96 | 5.90E-03 |
| ENSGALG00000034150 | CATHL3 | 2:4072545-4073720 | -3.40 | 6.18E-03 |
| ENSGALG00000049024 | CD7 | 18:3703058-3711715 | -1.53 | 6.22E-03 |
| ENSGALG00000029569 | ENSGALG00000029569 | 2:519057-520883 | -1.82 | 6.30E-03 |
| ENSGALG00000012407 | LRP1B | 7:31850143-32428239 | -1.14 | 6.33E-03 |
| ENSGALG00000054874 | ENSGALG00000054874 | 15:8227756-8228052 | -4.43 | 6.48E-03 |
| ENSGALG00000012421 | ARHGAP15 | 7:32698930-33027332 | -1.10 | 6.62E-03 |
| ENSGALG00000030712 | COL2A1 | 33:7292614-7310596 | -2.64 | 6.90E-03 |
| ENSGALG00000013481 | IKZF3 | 27:7069465-7091251 | -1.27 | 7.07E-03 |
| ENSGALG00000004771 | C1QB | 21:6073556-6075682 | -1.63 | 7.09E-03 |
| ENSGALG00000015362 | TRAT1 | 1:88912485-88924526 | -1.57 | 7.24E-03 |
| ENSGALG00000016261 | CYBB | 1:114457437-114478974 | -1.20 | 7.27E-03 |
| ENSGALG00000005263 | SOX8 | 14:6145956-6149345 | -1.12 | 7.43E-03 |
| ENSGALG00000025996 | ENSGALG00000025996 | 6:34319708-34339712 | -1.52 | 7.58E-03 |
| ENSGALG00000049486 | ENSGALG00000049486 | 15:8236440-8236721 | -5.41 | 7.71E-03 |
| ENSGALG00000048383 | ENSGALG00000048383 | 15:8232496-8232780 | -4.56 | 7.73E-03 |
| ENSGALG00000026038 | ERI2 | 14:15219039-15224073 | -1.63 | 7.77E-03 |
| ENSGALG00000036869 | NKX2-2 | 3:3523328-3524365 | -1.81 | 8.01E-03 |
| ENSGALG00000019696 | CATHL2 | 2:4074457-4076222 | -4.41 | 8.15E-03 |
| ENSGALG00000031735 | CD5 | 5:289584-293008 | -1.62 | 8.21E-03 |
| ENSGALG00000000466 | LCK | 23:5273969-5292034 | -1.02 | 8.22E-03 |
| ENSGALG00000006318 | IL21R | 14:7710463-7723251 | -2.04 | 8.49E-03 |
| ENSGALG00000032530 | SASH3 | 4:1634846-1637992 | -1.42 | 8.59E-03 |
| ENSGALG00000050218 | ENSGALG00000050218 | Z:35835-37390 | -4.11 | 8.62E-03 |
| ENSGALG00000043302 | LAT2 | 19:3007530-3016349 | -2.11 | 8.67E-03 |
| ENSGALG00000008656 | ICOS | 7:13376431-13388513 | -1.24 | 8.76E-03 |
| ENSGALG00000050947 | IFNL3 | 7:4572389-4573897 | -4.52 | 9.24E-03 |
| ENSGALG00000049186 | ENSGALG00000049186 | 15:8231361-8231642 | -4.92 | 9.32E-03 |
| ENSGALG00000052306 | ENSGALG00000052306 | 24:6481673-6491202 | -1.21 | 9.72E-03 |
| ENSGALG00000027765 | MMR1L1 | 2:19463738-19494337 | -1.39 | 1.00E-02 |
| ENSGALG00000015902 | CD8B | 4:85919110-85924985 | -2.06 | 1.01E-02 |
| ENSGALG00000002383 | CD72AG | Z:9066022-9069155 | -3.55 | 1.02E-02 |
| ENSGALG00000037558 | MEF2B | 28:3688008-3697772 | -2.46 | 1.03E-02 |
| ENSGALG00000052804 | ENSGALG00000052804 | 31:2417901-2418200 | -6.09 | 1.10E-02 |
| ENSGALG00000000544 | ENSGALG00000000544 | 26:1603818-1616373 | -1.77 | 1.10E-02 |
| ENSGALG00000008340 | FES | 10:20534450-20539528 | -1.28 | 1.12E-02 |
| ENSGALG00000005725 | CSF1R | 13:13980059-13997910 | -1.00 | 1.16E-02 |
| ENSGALG00000050967 | CHIR-AB1 | KZ626833.1:417501-615287 | -4.03 | 1.16E-02 |
| ENSGALG00000013086 | IKZF1 | 2:80659236-80730292 | -1.30 | 1.19E-02 |
| ENSGALG00000034427 | SCN4A | 27:4047358-4076818 | -1.43 | 1.21E-02 |
| ENSGALG00000008669 | CD28 | 7:13436135-13454946 | -1.98 | 1.26E-02 |
| ENSGALG00000052552 | ENSGALG00000052552 | 30:614511-621897 | -1.72 | 1.30E-02 |
| ENSGALG00000052959 | ENSGALG00000052959 | 1:79025623-79038498 | -1.47 | 1.30E-02 |
| ENSGALG00000009479 | ENSGALG00000009479 | 2:22911382-22916121 | -1.61 | 1.31E-02 |
| ENSGALG00000006323 | LECT2 | 13:15913091-15918063 | -3.18 | 1.32E-02 |
| ENSGALG00000016173 | UBASH3A | 1:110728999-110753448 | -1.43 | 1.33E-02 |
| ENSGALG00000045115 | ENSGALG00000045115 | 30:595215-599886 | -1.63 | 1.34E-02 |
| ENSGALG00000038559 | CD8A | 4:85657238-85736578 | -1.68 | 1.41E-02 |
| ENSGALG00000050946 | ENSGALG00000050946 | 31:4750522-4752709 | -1.57 | 1.43E-02 |
| ENSGALG00000012080 | ENSGALG00000012080 | 2:46175212-46178881 | -2.09 | 1.50E-02 |
| ENSGALG00000003217 | LITAF | 14:1329782-1337607 | -1.36 | 1.51E-02 |
| ENSGALG00000052542 | ENSGALG00000052542 | 31:2371882-2372184 | -5.01 | 1.51E-02 |
| ENSGALG00000052133 | ENSGALG00000052133 | 5:30048598-30067217 | -1.24 | 1.53E-02 |
| ENSGALG00000040716 | PRLH | 7:4813216-4814151 | -3.21 | 1.56E-02 |
| ENSGALG00000038000 | CX3CR1 | 2:44330678-44340445 | -1.50 | 1.60E-02 |
| ENSGALG00000013079 | ENSGALG00000013079 | 2:80073338-80135176 | -3.52 | 1.62E-02 |
| ENSGALG00000025881 | ENSGALG00000025881 | 1:51541954-51569755 | -1.22 | 1.62E-02 |
| ENSGALG00000045940 | ENSGALG00000045940 | 3:105078987-105090249 | -1.40 | 1.64E-02 |
| ENSGALG00000048867 | ENSGALG00000048867 | 12:17743400-17774585 | -2.25 | 1.69E-02 |
| ENSGALG00000001149 | ADGRG5 | 11:543433-548011 | -1.49 | 1.75E-02 |
| ENSGALG00000051595 | ENSGALG00000051595 | 31:5815164-5817830 | -4.22 | 1.77E-02 |
| ENSGALG00000030660 | DNAJC5B | 2:114919024-114955857 | -1.39 | 1.80E-02 |
| ENSGALG00000049536 | ENSGALG00000049536 | 1:197028549-197031106 | -1.35 | 1.83E-02 |
| ENSGALG00000035194 | ARHGAP25 | 22:437194-453936 | -1.08 | 1.86E-02 |
| ENSGALG00000014750 | ENSGALG00000014750 | 1:78771732-78773515 | -1.99 | 1.86E-02 |
| ENSGALG00000007713 | CD300LG | 18:10410168-10414906 | -1.35 | 1.86E-02 |
| ENSGALG00000048283 | ENSGALG00000048283 | 15:8220872-8221078 | -5.09 | 1.87E-02 |
| ENSGALG00000030684 | ENSGALG00000030684 | 15:9086579-9099994 | -1.27 | 1.87E-02 |
| ENSGALG00000006402 | NRROS | 9:4855404-4859977 | -1.24 | 1.88E-02 |
| ENSGALG00000047440 | ENSGALG00000047440 | 31:2376712-2377014 | -4.69 | 1.89E-02 |
| ENSGALG00000035818 | ENSGALG00000035818 | 33:4895192-4926729 | -1.79 | 1.90E-02 |
| ENSGALG00000049682 | CHIR-B2 | 31:4380382-4383366 | -3.75 | 1.92E-02 |
| ENSGALG00000055067 | ENSGALG00000055067 | 1:180854989-180866070 | -1.29 | 1.96E-02 |
| ENSGALG00000045762 | ENSGALG00000045762 | 1:49487070-49493031 | -1.58 | 1.96E-02 |
| ENSGALG00000031978 | TARP | 2:49030132-49126360 | -1.16 | 1.99E-02 |
| ENSGALG00000026663 | CX3CL1 | 11:771897-778309 | -1.01 | 2.00E-02 |
| ENSGALG00000053039 | ENSGALG00000053039 | 1:189756527-189768663 | -3.42 | 2.04E-02 |
| ENSGALG00000044071 | ABI3 | 27:6041139-6044622 | -1.07 | 2.09E-02 |
| ENSGALG00000002982 | SLC2A6 | 17:7235916-7240174 | -1.49 | 2.10E-02 |
| ENSGALG00000000081 | IL4I1 | 16:2367597-2478250 | -2.04 | 2.15E-02 |
| ENSGALG00000003076 | FASLG | 8:4846877-4850856 | -2.14 | 2.17E-02 |
| ENSGALG00000012801 | LY86 | 2:64889269-64914270 | -1.31 | 2.17E-02 |
| ENSGALG00000015662 | ENSGALG00000015662 | 1:99391578-99407800 | -2.09 | 2.18E-02 |
| ENSGALG00000046409 | ENSGALG00000046409 | 5:45784600-45790095 | -1.81 | 2.21E-02 |
| ENSGALG00000051069 | ENSGALG00000051069 | 12:2152920-2160926 | -1.51 | 2.25E-02 |
| ENSGALG00000046393 | ENSGALG00000046393 | 25:2461040-2465729 | -1.64 | 2.27E-02 |
| ENSGALG00000054411 | ENSGALG00000054411 | 6:19441997-19461945 | -2.35 | 2.34E-02 |
| ENSGALG00000001234 | CETP | 11:623460-629266 | -2.07 | 2.39E-02 |
| ENSGALG00000040136 | PSTPIP2 | Z:2160803-2176161 | -1.60 | 2.40E-02 |
| ENSGALG00000045581 | ENSGALG00000045581 | 2:505017-507099 | -1.19 | 2.44E-02 |
| ENSGALG00000037599 | ENSGALG00000037599 | 25:3312351-3314646 | -3.74 | 2.46E-02 |
| ENSGALG00000029669 | C1QC | 21:6076987-6078857 | -1.37 | 2.51E-02 |
| ENSGALG00000004336 | INPPL1 | 4:1229311-1237808 | -1.42 | 2.52E-02 |
| ENSGALG00000049823 | ENSGALG00000049823 | 31:2383549-2383851 | -5.83 | 2.56E-02 |
| ENSGALG00000016400 | RSAD2 | 3:94991681-95016338 | -1.17 | 2.58E-02 |
| ENSGALG00000049402 | ENSGALG00000049402 | 26:2642206-2646189 | -1.46 | 2.59E-02 |
| ENSGALG00000047852 | ENSGALG00000047852 | 4:15886725-16039540 | -1.33 | 2.59E-02 |
| ENSGALG00000005212 | GHRHR | 2:1238715-1257265 | -1.38 | 2.59E-02 |
| ENSGALG00000046795 | RF00017 | 5:57738304-57738603 | -2.27 | 2.61E-02 |
| ENSGALG00000003554 | OPTC | 26:5176373-5179960 | -1.16 | 2.64E-02 |
| ENSGALG00000047506 | ENSGALG00000047506 | 15:8235121-8235417 | -4.14 | 2.64E-02 |
| ENSGALG00000043064 | EXFABP | 17:865358-868425 | -1.15 | 2.65E-02 |
| ENSGALG00000021884 | ENSGALG00000021884 | 28:3532747-3534622 | -1.73 | 2.68E-02 |
| ENSGALG00000049221 | ENSGALG00000049221 | 7:13468414-13472570 | -1.64 | 2.68E-02 |
| ENSGALG00000037642 | SLC23A1 | 13:3188490-3191561 | -2.47 | 2.69E-02 |
| ENSGALG00000011646 | PLD4 | 5:51984130-51996689 | -1.25 | 2.72E-02 |
| ENSGALG00000029857 | ENSGALG00000029857 | 2:4138557-4140735 | -1.29 | 2.73E-02 |
| ENSGALG00000030291 | IRF10 | 20:10063682-10066572 | -1.04 | 2.82E-02 |
| ENSGALG00000013218 | C3AR1 | 1:66793430-66796768 | -1.52 | 2.83E-02 |
| ENSGALG00000021355 | ENSGALG00000021355 | Z:9015759-9024777 | -1.29 | 2.89E-02 |
| ENSGALG00000033700 | CA3A | 2:122678180-122689749 | -1.88 | 2.89E-02 |
| ENSGALG00000048531 | ENSGALG00000048531 | 31:2395480-2395779 | -4.17 | 2.92E-02 |
| ENSGALG00000047851 | ENSGALG00000047851 | 1:113354652-113362393 | -3.16 | 2.97E-02 |
| ENSGALG00000051025 | ENSGALG00000051025 | 4:11543820-11556315 | -1.10 | 2.99E-02 |
| ENSGALG00000028519 | CCR10 | 27:7819486-7821867 | -3.35 | 3.07E-02 |
| ENSGALG00000046032 | CD83 | 2:60692920-60705622 | -1.16 | 3.08E-02 |
| ENSGALG00000045847 | GRXCR2 | 13:18466510-18483465 | -1.20 | 3.15E-02 |
| ENSGALG00000051110 | ENSGALG00000051110 | 10:16871683-16872843 | -3.08 | 3.22E-02 |
| ENSGALG00000037077 | CCR5 | 2:42617494-42623187 | -1.38 | 3.25E-02 |
| ENSGALG00000024490 | ENSGALG00000024490 | 4:69443374-69469660 | -1.75 | 3.33E-02 |
| ENSGALG00000052896 | ENSGALG00000052896 | 12:3651744-3652932 | -1.22 | 3.35E-02 |
| ENSGALG00000006394 | ENSGALG00000006394 | 14:7726359-7738340 | -1.96 | 3.36E-02 |
| ENSGALG00000051140 | ENSGALG00000051140 | 4:14530005-14616390 | -1.29 | 3.37E-02 |
| ENSGALG00000038069 | CCR8 | 2:44316108-44319487 | -1.73 | 3.38E-02 |
| ENSGALG00000006480 | TCF7 | 13:16620708-16679055 | -1.37 | 3.38E-02 |
| ENSGALG00000052537 | ENSGALG00000052537 | 8:4097967-4100381 | -1.83 | 3.49E-02 |
| ENSGALG00000025810 | CCNB3 | 4:1834030-1837284 | -1.45 | 3.49E-02 |
| ENSGALG00000004700 | NCF2 | 8:7994109-8002276 | -1.32 | 3.51E-02 |
| ENSGALG00000008660 | CST7 | 3:15917270-15922608 | -1.36 | 3.51E-02 |
| ENSGALG00000013372 | IL7R | Z:10886978-10900701 | -1.13 | 3.56E-02 |
| ENSGALG00000051485 | ENSGALG00000051485 | 31:2429043-2429341 | -5.34 | 3.63E-02 |
| ENSGALG00000023760 | CHIA | 26:5081076-5100133 | -2.37 | 3.69E-02 |
| ENSGALG00000000274 | ENSGALG00000000274 | 27:4079597-4087573 | -2.95 | 3.77E-02 |
| ENSGALG00000016788 | IL18RAP | 1:135229034-135244807 | -1.94 | 3.79E-02 |
| ENSGALG00000026091 | CACNG4 | 18:7236970-7272838 | -1.28 | 3.81E-02 |
| ENSGALG00000002102 | ENSGALG00000002102 | 10:1841767-1843695 | -1.73 | 3.85E-02 |
| ENSGALG00000054174 | ENSGALG00000054174 | 28:1370732-1372658 | -2.28 | 3.89E-02 |
| ENSGALG00000047130 | ENSGALG00000047130 | 20:9781126-9784960 | -1.19 | 3.89E-02 |
| ENSGALG00000047181 | ENSGALG00000047181 | 2:120371810-120421763 | -1.57 | 3.90E-02 |
| ENSGALG00000034478 | CCL4 | 19:576608-578177 | -1.53 | 3.95E-02 |
| ENSGALG00000053738 | ENSGALG00000053738 | 2:44322489-44334234 | -2.11 | 3.97E-02 |
| ENSGALG00000048508 | ENSGALG00000048508 | 30:631147-635961 | -2.13 | 3.98E-02 |
| ENSGALG00000003911 | CACNG5 | 18:7295811-7307917 | -1.01 | 3.98E-02 |
| ENSGALG00000031826 | MYOT | 13:15119631-15132751 | -1.86 | 4.02E-02 |
| ENSGALG00000035589 | ENSGALG00000035589 | 19:9178482-9181084 | -1.25 | 4.17E-02 |
| ENSGALG00000004252 | ENSGALG00000004252 | 28:5030693-5034171 | -1.16 | 4.21E-02 |
| ENSGALG00000050332 | ENSGALG00000050332 | 14:2745676-2753557 | -1.24 | 4.25E-02 |
| ENSGALG00000007546 | ENSGALG00000007546 | 4:11345577-11367340 | -1.19 | 4.27E-02 |
| ENSGALG00000049726 | ENSGALG00000049726 | 31:2398508-2398810 | -4.09 | 4.27E-02 |
| ENSGALG00000013869 | IL20RA | 3:54956242-54977305 | -1.39 | 4.29E-02 |
| ENSGALG00000023818 | HSPB9 | 27:7654604-7655550 | -1.61 | 4.29E-02 |
| ENSGALG00000047247 | ENSGALG00000047247 | 1:93310597-93323987 | -1.63 | 4.31E-02 |
| ENSGALG00000043904 | SH3RF2 | 13:18478205-18497381 | -1.10 | 4.33E-02 |
| ENSGALG00000047627 | ENSGALG00000047627 | 16:2082890-2084768 | -1.37 | 4.42E-02 |
| ENSGALG00000007675 | CXCR5 | 24:5596881-5602957 | -2.04 | 4.54E-02 |
| ENSGALG00000023780 | TREM-B2 | 26:4755127-4761551 | -1.22 | 4.57E-02 |
| ENSGALG00000048761 | ENSGALG00000048761 | 31:2380719-2381009 | -4.72 | 4.60E-02 |
| ENSGALG00000011961 | TNIP3 | 4:54109012-54173032 | -1.15 | 4.67E-02 |
| ENSGALG00000023709 | TOR1BL | 17:5990121-5992857 | -1.56 | 4.80E-02 |
| ENSGALG00000044339 | ENSGALG00000044339 | 24:5459531-5465244 | -1.09 | 4.88E-02 |
| ENSGALG00000039978 | SLC4A1 | 27:5419090-5434440 | -1.40 | 4.91E-02 |
| ENSGALG00000006527 | CRTAM | 24:3151297-3163767 | -1.06 | 4.94E-02 |
| ENSGALG00000047114 | ENSGALG00000047114 | 31:2434641-2434943 | -4.16 | 4.95E-02 |
| ENSGALG00000015434 | GRIK2 | 3:70157498-70521359 | 1.39 | 1.60E-06 |
| ENSGALG00000004083 | GRIA1 | 13:13030976-13139199 | 1.53 | 3.34E-05 |
| ENSGALG00000010909 | PRKG2 | 4:45615049-45636930 | 1.44 | 7.51E-05 |
| ENSGALG00000032028 | SERTM1 | 1:173838414-173854684 | 1.18 | 1.18E-04 |
| ENSGALG00000012200 | GCH1 | 5:56530534-56547633 | 1.05 | 2.50E-04 |
| ENSGALG00000013154 | SLCO1C1 | 1:65137008-65158246 | 3.68 | 3.62E-04 |
| ENSGALG00000010470 | TCERG1L | 6:35538901-35604326 | 1.05 | 5.10E-04 |
| ENSGALG00000005140 | RRAD | 11:11211167-11216791 | 1.20 | 6.69E-04 |
| ENSGALG00000039145 | CA8 | 2:112701443-112753254 | 1.76 | 7.29E-04 |
| ENSGALG00000036204 | ENSGALG00000036204 | 3:72293898-72433758 | 1.15 | 7.44E-04 |
| ENSGALG00000016757 | TSGA10 | 1:133775253-133793015 | 1.65 | 9.14E-04 |
| ENSGALG00000054272 | ENSGALG00000054272 | 14:9150908-9162220 | 2.04 | 1.21E-03 |
| ENSGALG00000003553 | ABCA12 | 7:4266278-4345086 | 1.97 | 1.51E-03 |
| ENSGALG00000040567 | AVPR1A | 1:33419044-33421641 | 1.38 | 1.90E-03 |
| ENSGALG00000007070 | ENSGALG00000007070 | 14:9166630-9185734 | 1.79 | 1.91E-03 |
| ENSGALG00000054951 | ENSGALG00000054951 | 18:322440-327016 | 2.95 | 2.26E-03 |
| ENSGALG00000054878 | ENSGALG00000054878 | 4:9528971-9531665 | 3.06 | 2.66E-03 |
| ENSGALG00000029170 | NPBWR2 | 20:9240792-9260447 | 1.07 | 2.81E-03 |
| ENSGALG00000050315 | CLC2DL3 | 1:216908-303711 | 1.13 | 3.43E-03 |
| ENSGALG00000013117 | PIK3C2G | 1:63981655-64179076 | 1.05 | 3.57E-03 |
| ENSGALG00000054601 | ENSGALG00000054601 | 8:474054-507786 | 1.73 | 3.61E-03 |
| ENSGALG00000001109 | GTSF1 | 27:3444203-3455470 | 1.55 | 3.64E-03 |
| ENSGALG00000002112 | CSF3R | 23:4160678-4166175 | 1.34 | 4.05E-03 |
| ENSGALG00000027192 | NTS | 1:42207171-42220099 | 1.69 | 4.23E-03 |
| ENSGALG00000054605 | ENSGALG00000054605 | 14:11277460-11280513 | 3.10 | 4.23E-03 |
| ENSGALG00000028037 | FOS | 5:38430793-38433702 | 1.61 | 4.26E-03 |
| ENSGALG00000031423 | DMRTA2 | 8:24139262-24140464 | 1.19 | 4.62E-03 |
| ENSGALG00000037402 | CTGF | 3:57245588-57248925 | 1.06 | 4.93E-03 |
| ENSGALG00000001716 | C10H15ORF59 | 10:2728829-2736012 | 1.27 | 6.46E-03 |
| ENSGALG00000015244 | TBX19 | 1:85209232-85223485 | 1.09 | 6.68E-03 |
| ENSGALG00000000371 | CRHR1 | 27:4646659-4670553 | 1.20 | 6.73E-03 |
| ENSGALG00000054946 | ENSGALG00000054946 | Z:57311687-57318534 | 1.75 | 7.28E-03 |
| ENSGALG00000015838 | MRAP2 | 3:77520011-77538966 | 1.78 | 7.65E-03 |
| ENSGALG00000016600 | POMC | 3:105372181-105388173 | 1.11 | 8.18E-03 |
| ENSGALG00000013782 | CDH7 | 2:95468521-95545982 | 2.08 | 8.68E-03 |
| ENSGALG00000048415 | ENSGALG00000048415 | 5:46381063-46418607 | 1.09 | 9.23E-03 |
| ENSGALG00000016086 | EGR4 | 4:89672386-89673546 | 3.30 | 9.86E-03 |
| ENSGALG00000000769 | RAB7B | 26:2349209-2352491 | 1.06 | 1.02E-02 |
| ENSGALG00000010719 | NEIL3 | 4:42233732-42250523 | 1.50 | 1.03E-02 |
| ENSGALG00000008661 | CYR61 | 8:16587842-16590293 | 1.13 | 1.05E-02 |
| ENSGALG00000025955 | gga-mir-3523 | 13:9772409-9772574 | 4.09 | 1.06E-02 |
| ENSGALG00000054009 | ENSGALG00000054009 | 1:18792907-18810553 | 2.06 | 1.11E-02 |
| ENSGALG00000049733 | ENSGALG00000049733 | 5:33745774-33752621 | 1.33 | 1.13E-02 |
| ENSGALG00000012873 | SERPINB5 | 2:67749398-67762854 | 1.62 | 1.21E-02 |
| ENSGALG00000051373 | ENSGALG00000051373 | 4:9528445-9531664 | 3.24 | 1.28E-02 |
| ENSGALG00000009219 | C3H1orf95 | 3:17045413-17078471 | 1.04 | 1.31E-02 |
| ENSGALG00000035351 | ENSGALG00000035351 | 1:141461074-141463479 | 4.10 | 1.32E-02 |
| ENSGALG00000015720 | CHODL | 1:100912368-100941544 | 2.96 | 1.32E-02 |
| ENSGALG00000037997 | ENSGALG00000037997 | 2:128211045-128243190 | 1.46 | 1.41E-02 |
| ENSGALG00000006629 | RBP4A | 6:21325448-21360467 | 1.42 | 1.46E-02 |
| ENSGALG00000035619 | MTTP | 4:60028589-60056286 | 3.82 | 1.49E-02 |
| ENSGALG00000017046 | POSTN | 1:173372443-173406135 | 1.71 | 1.50E-02 |
| ENSGALG00000015494 | HTR1F | 1:93982791-93983891 | 1.80 | 1.62E-02 |
| ENSGALG00000001437 | NTM | 24:1887297-2185646 | 1.08 | 1.66E-02 |
| ENSGALG00000040761 | DRD4 | 5:785916-791818 | 1.24 | 1.66E-02 |
| ENSGALG00000052522 | ENSGALG00000052522 | 1:81820856-81823870 | 3.76 | 1.71E-02 |
| ENSGALG00000008135 | SATB2 | 7:10794292-10924557 | 2.39 | 1.71E-02 |
| ENSGALG00000050186 | ENSGALG00000050186 | 3:39106153-39109434 | 3.04 | 2.03E-02 |
| ENSGALG00000054405 | ENSGALG00000054405 | 1:141606019-141610430 | 3.53 | 2.09E-02 |
| ENSGALG00000042705 | KCNK3 | 3:105215798-105226949 | 1.12 | 2.11E-02 |
| ENSGALG00000053164 | ENSGALG00000053164 | 2:25649973-25652063 | 2.64 | 2.22E-02 |
| ENSGALG00000007785 | PROK2 | 12:16703105-16707773 | 1.82 | 2.27E-02 |
| ENSGALG00000007669 | EGR1 | 13:18949948-18952753 | 1.28 | 2.29E-02 |
| ENSGALG00000003013 | ENSGALG00000003013 | 6:9115242-9166926 | 1.06 | 2.33E-02 |
| ENSGALG00000040436 | GAD2 | 2:16267297-16299782 | 1.84 | 2.36E-02 |
| ENSGALG00000038740 | AMY2A | 8:440100-444521 | 1.34 | 2.41E-02 |
| ENSGALG00000038787 | ADRA1D | 4:88341840-88377988 | 1.09 | 2.48E-02 |
| ENSGALG00000050807 | ENSGALG00000050807 | 6:4575545-4595569 | 1.89 | 2.51E-02 |
| ENSGALG00000002594 | TFPI | 7:999492-1117976 | 1.01 | 2.51E-02 |
| ENSGALG00000045264 | ENSGALG00000045264 | 10:2776348-2783810 | 1.47 | 2.62E-02 |
| ENSGALG00000051425 | ENSGALG00000051425 | 17:7884609-7893584 | 1.35 | 2.70E-02 |
| ENSGALG00000043931 | ENSGALG00000043931 | 1:141586779-141588257 | 3.14 | 2.70E-02 |
| ENSGALG00000015753 | ENSGALG00000015753 | Z:67243630-67276948 | 1.85 | 2.72E-02 |
| ENSGALG00000014979 | FRK | 3:64064317-64109976 | 1.26 | 2.77E-02 |
| ENSGALG00000001934 | OPN4 | 6:3059913-3082527 | 1.63 | 3.00E-02 |
| ENSGALG00000053800 | ENSGALG00000053800 | 1:37567265-37589352 | 3.15 | 3.05E-02 |
| ENSGALG00000047026 | ENSGALG00000047026 | 1:1825448-1843919 | 1.67 | 3.06E-02 |
| ENSGALG00000052212 | ENSGALG00000052212 | 3:38342260-38362857 | 1.05 | 3.08E-02 |
| ENSGALG00000053949 | ENSGALG00000053949 | 2:53115962-53167531 | 3.81 | 3.13E-02 |
| ENSGALG00000037870 | ENSGALG00000037870 | 7:23202420-23229758 | 1.84 | 3.22E-02 |
| ENSGALG00000030801 | CCK1R | 4:73203371-73210227 | 1.29 | 3.25E-02 |
| ENSGALG00000031689 | ENSGALG00000031689 | 6:22900942-22919904 | 1.04 | 3.31E-02 |
| ENSGALG00000053159 | ENSGALG00000053159 | 17:8354154-8363823 | 1.47 | 3.48E-02 |
| ENSGALG00000048096 | ENSGALG00000048096 | 2:47032286-47070696 | 1.94 | 3.57E-02 |
| ENSGALG00000007980 | KCNG1 | 20:13362490-13370389 | 1.16 | 3.62E-02 |
| ENSGALG00000049422 | ATF3 | 3:21942839-21967759 | 1.01 | 3.65E-02 |
| ENSGALG00000002098 | GRIK3 | 23:4000662-4069929 | 1.10 | 3.67E-02 |
| ENSGALG00000028720 | ENSGALG00000028720 | 1:99545973-99575148 | 3.70 | 3.70E-02 |
| ENSGALG00000040971 | KROX20 | 6:8758662-8761245 | 1.44 | 3.79E-02 |
| ENSGALG00000047746 | ENSGALG00000047746 | 5:33745802-33940089 | 1.80 | 3.81E-02 |
| ENSGALG00000049525 | ENSGALG00000049525 | 3:68035569-68079617 | 1.08 | 3.91E-02 |
| ENSGALG00000005318 | OVCH2 | 5:7676131-7692468 | 1.42 | 4.06E-02 |
| ENSGALG00000047657 | ENSGALG00000047657 | 2:89336026-89349197 | 1.71 | 4.09E-02 |
| ENSGALG00000047759 | ENSGALG00000047759 | 8:1085113-1107488 | 3.23 | 4.12E-02 |
| ENSGALG00000041950 | ENSGALG00000041950 | 20:9213984-9217305 | 1.17 | 4.22E-02 |
| ENSGALG00000027144 | PTF1A | 2:17415549-17417320 | 1.50 | 4.30E-02 |
| ENSGALG00000053154 | ENSGALG00000053154 | 1:54982805-55044149 | 2.85 | 4.35E-02 |
| ENSGALG00000050994 | ENSGALG00000050994 | 2:99761554-99883697 | 1.84 | 4.38E-02 |
| ENSGALG00000055007 | ENSGALG00000055007 | 5:9269302-9279385 | 2.83 | 4.39E-02 |
| ENSGALG00000015028 | RLN3 | Z:27624861-27627531 | 2.07 | 4.46E-02 |
| ENSGALG00000054402 | ENSGALG00000054402 | KZ626839.1:361032-374943 | 1.32 | 4.51E-02 |
| ENSGALG00000049107 | ENSGALG00000049107 | 1:90247614-90265685 | 1.41 | 4.59E-02 |
| ENSGALG00000051532 | ENSGALG00000051532 | 2:88628887-88648966 | 3.40 | 4.71E-02 |
| ENSGALG00000050650 | ENSGALG00000050650 | 22:4194409-4207430 | 1.23 | 4.74E-02 |
| ENSGALG00000047100 | ENSGALG00000047100 | 3:11257288-11313086 | 2.47 | 4.76E-02 |
| ENSGALG00000042963 | ENSGALG00000042963 | 27:2222448-2250786 | 2.24 | 4.83E-02 |
| ENSGALG00000050137 | ENSGALG00000050137 | 9:21999941-22085829 | 2.39 | 4.87E-02 |
| ENSGALG00000048291 | ENSGALG00000048291 | 3:39104462-39106051 | 3.24 | 4.93E-02 |
| ENSGALG00000055015 | ENSGALG00000055015 | 20:11742726-11934392 | 3.35 | 4.94E-02 |
| ENSGALG00000052818 | ENSGALG00000052818 | 11:12899315-12901183 | 2.22 | 4.99E-02 |

**Supplementary Table S7. Functional annotation of DEGs by gene ontology (GO) analysis.**

| **Category** | **Term** | **genes** | **FDR** |
| --- | --- | --- | --- |
| GOTERM_BP | immune system process | 68 | 5.20E-25 |
| GOTERM_BP | immune response | 47 | 1.60E-21 |
| GOTERM_BP | response to stimulus | 102 | 8.40E-10 |
| GOTERM_BP | defense response | 33 | 2.50E-08 |
| GOTERM_BP | hemopoiesis | 27 | 7.20E-08 |
| GOTERM_BP | positive regulation of immune system process | 25 | 7.20E-08 |
| GOTERM_BP | regulation of immune system process | 31 | 1.40E-07 |
| GOTERM_BP | hematopoietic or lymphoid organ development | 27 | 2.00E-07 |
| GOTERM_BP | leukocyte activation | 24 | 2.10E-07 |
| GOTERM_BP | immune system development | 27 | 4.90E-07 |
| GOTERM_BP | response to external stimulus | 40 | 9.40E-07 |
| GOTERM_BP | leukocyte differentiation | 20 | 9.90E-07 |
| GOTERM_BP | immune effector process | 21 | 2.00E-06 |
| GOTERM_BP | cell activation | 24 | 4.60E-06 |
| GOTERM_BP | leukocyte cell-cell adhesion | 18 | 5.60E-06 |
| GOTERM_BP | innate immune response | 18 | 5.70E-06 |
| GOTERM_BP | regulation of immune response | 19 | 9.00E-06 |
| GOTERM_BP | lymphocyte activation | 20 | 1.10E-05 |
| GOTERM_BP | lymphocyte differentiation | 15 | 2.90E-05 |
| GOTERM_BP | T cell aggregation | 16 | 3.00E-05 |
| GOTERM_BP | T cell activation | 16 | 3.00E-05 |
| GOTERM_BP | lymphocyte aggregation | 16 | 3.10E-05 |
| GOTERM_BP | leukocyte aggregation | 16 | 3.60E-05 |
| GOTERM_BP | activation of immune response | 14 | 3.70E-05 |
| GOTERM_BP | cytokine production | 18 | 4.20E-05 |
| GOTERM_BP | positive regulation of immune response | 16 | 4.70E-05 |
| GOTERM_BP | regulation of cytokine production | 17 | 5.00E-05 |
| GOTERM_BP | T cell differentiation | 12 | 5.60E-05 |
| GOTERM_BP | leukocyte chemotaxis | 11 | 8.50E-05 |
| GOTERM_BP | myeloid leukocyte migration | 10 | 1.00E-04 |
| GOTERM_BP | leukocyte mediated immunity | 11 | 2.40E-04 |
| GOTERM_BP | cell chemotaxis | 12 | 2.70E-04 |
| GOTERM_BP | leukocyte migration | 12 | 2.70E-04 |
| GOTERM_BP | positive regulation of biological process | 66 | 2.80E-04 |
| GOTERM_BP | immune response-activating signal transduction | 12 | 2.90E-04 |
| GOTERM_BP | chemotaxis | 17 | 2.90E-04 |
| GOTERM_BP | single organismal cell-cell adhesion | 19 | 2.90E-04 |
| GOTERM_BP | taxis | 17 | 2.90E-04 |
| GOTERM_BP | regulation of hemopoiesis | 13 | 3.70E-04 |
| GOTERM_BP | immune response-regulating signaling pathway | 12 | 3.70E-04 |
| GOTERM_BP | adaptive immune response | 11 | 6.80E-04 |
| GOTERM_BP | neutrophil chemotaxis | 7 | 9.50E-04 |
| GOTERM_BP | single organism cell adhesion | 19 | 1.00E-03 |
| GOTERM_BP | response to stress | 46 | 1.10E-03 |
| GOTERM_BP | cell surface receptor signaling pathway | 37 | 1.20E-03 |
| GOTERM_BP | alpha-beta T cell activation | 8 | 1.20E-03 |
| GOTERM_BP | biological regulation | 110 | 1.20E-03 |
| GOTERM_BP | positive regulation of hemopoiesis | 9 | 1.30E-03 |
| GOTERM_BP | neutrophil migration | 7 | 1.30E-03 |
| GOTERM_BP | positive regulation of cellular process | 59 | 1.40E-03 |
| GOTERM_BP | response to biotic stimulus | 18 | 1.70E-03 |
| GOTERM_BP | cell-cell adhesion | 20 | 2.40E-03 |
| GOTERM_BP | regulation of multicellular organismal process | 39 | 2.40E-03 |
| GOTERM_BP | single organism signaling | 66 | 2.40E-03 |
| GOTERM_BP | granulocyte chemotaxis | 7 | 2.40E-03 |
| GOTERM_BP | myeloid leukocyte activation | 8 | 2.40E-03 |
| GOTERM_BP | immune response-activating cell surface receptor signaling pathway | 8 | 2.60E-03 |
| GOTERM_BP | signal transduction | 62 | 2.60E-03 |
| GOTERM_BP | cell adhesion | 26 | 2.60E-03 |
| GOTERM_BP | biological adhesion | 26 | 2.70E-03 |
| GOTERM_BP | response to external biotic stimulus | 17 | 2.70E-03 |
| GOTERM_BP | response to other organism | 17 | 2.70E-03 |
| GOTERM_BP | granulocyte migration | 7 | 2.70E-03 |
| GOTERM_BP | cellular response to stimulus | 73 | 2.70E-03 |
| GOTERM_BP | signaling | 66 | 2.70E-03 |
| GOTERM_BP | immune response-regulating cell surface receptor signaling pathway | 8 | 3.00E-03 |
| GOTERM_BP | positive regulation of response to stimulus | 30 | 3.10E-03 |
| GOTERM_BP | cell communication | 66 | 3.20E-03 |
| GOTERM_BP | regulation of biological process | 103 | 3.30E-03 |
| GOTERM_BP | regulation of myeloid cell differentiation | 9 | 4.90E-03 |
| GOTERM_BP | regulation of leukocyte mediated immunity | 7 | 5.30E-03 |
| GOTERM_BP | lymphocyte mediated immunity | 8 | 5.50E-03 |
| GOTERM_BP | response to chemical | 43 | 5.60E-03 |
| GOTERM_BP | positive regulation of multicellular organismal process | 25 | 5.80E-03 |
| GOTERM_BP | single-multicellular organism process | 66 | 5.80E-03 |
| GOTERM_BP | positive regulation of response to external stimulus | 9 | 6.80E-03 |
| GOTERM_BP | regulation of cellular process | 98 | 7.60E-03 |
| GOTERM_BP | multicellular organismal process | 70 | 7.90E-03 |
| GOTERM_BP | positive regulation of leukocyte differentiation | 7 | 8.70E-03 |
| GOTERM_BP | regulation of leukocyte differentiation | 9 | 8.90E-03 |
| GOTERM_BP | adaptive immune response based on somatic recombination of immune receptors built from immunoglobulin superfamily domains | 8 | 9.80E-03 |
| GOTERM_BP | regulation of leukocyte cell-cell adhesion | 9 | 9.80E-03 |
| GOTERM_BP | localization of cell | 24 | 1.10E-02 |
| GOTERM_BP | locomotion | 26 | 1.20E-02 |
| GOTERM_BP | regulation of leukocyte activation | 11 | 1.30E-02 |
| GOTERM_BP | regulation of cAMP biosynthetic process | 6 | 1.30E-02 |
| GOTERM_BP | myeloid cell differentiation | 11 | 1.30E-02 |
| GOTERM_BP | animal organ development | 41 | 1.30E-02 |
| GOTERM_BP | positive regulation of myeloid cell differentiation | 6 | 1.50E-02 |
| GOTERM_BP | cell migration | 22 | 1.60E-02 |
| GOTERM_BP | alpha-beta T cell differentiation | 6 | 1.60E-02 |
| GOTERM_BP | positive regulation of cytokine production | 10 | 1.60E-02 |
| GOTERM_BP | positive regulation of T cell activation | 7 | 1.70E-02 |
| GOTERM_BP | myeloid cell activation involved in immune response | 5 | 1.90E-02 |
| GOTERM_BP | leukocyte activation involved in immune response | 8 | 1.90E-02 |
| GOTERM_BP | regulation of lymphocyte activation | 10 | 1.90E-02 |
| GOTERM_BP | regulation of cAMP metabolic process | 6 | 1.90E-02 |
| GOTERM_BP | inflammatory response | 12 | 2.00E-02 |
| GOTERM_BP | cell activation involved in immune response | 8 | 2.00E-02 |
| GOTERM_BP | positive regulation of leukocyte cell-cell adhesion | 7 | 2.00E-02 |
| GOTERM_BP | positive regulation of cAMP biosynthetic process | 5 | 2.00E-02 |
| GOTERM_BP | regulation of cell activation | 11 | 2.10E-02 |
| GOTERM_BP | cell motility | 23 | 2.10E-02 |
| GOTERM_BP | homeostatic process | 26 | 2.10E-02 |
| GOTERM_BP | cAMP biosynthetic process | 6 | 2.10E-02 |
| GOTERM_BP | lymphocyte proliferation | 9 | 2.10E-02 |
| GOTERM_BP | mononuclear cell proliferation | 9 | 2.10E-02 |
| GOTERM_BP | positive regulation of cAMP metabolic process | 5 | 2.20E-02 |
| GOTERM_BP | defense response to other organism | 11 | 2.40E-02 |
| GOTERM_BP | regulation of cyclic nucleotide biosynthetic process | 6 | 2.40E-02 |
| GOTERM_BP | T cell mediated immunity | 5 | 2.40E-02 |
| GOTERM_BP | regulation of T cell activation | 8 | 2.60E-02 |
| GOTERM_BP | leukocyte proliferation | 9 | 2.70E-02 |
| GOTERM_BP | regulation of nucleotide biosynthetic process | 6 | 3.00E-02 |
| GOTERM_BP | regulation of purine nucleotide biosynthetic process | 6 | 3.00E-02 |
| GOTERM_BP | cAMP metabolic process | 6 | 3.20E-02 |
| GOTERM_BP | positive regulation of leukocyte chemotaxis | 5 | 3.70E-02 |
| GOTERM_BP | humoral immune response | 6 | 3.70E-02 |
| GOTERM_BP | regulation of response to stimulus | 42 | 3.80E-02 |
| GOTERM_BP | positive regulation of cell differentiation | 16 | 3.90E-02 |
| GOTERM_BP | positive regulation of cell-cell adhesion | 7 | 4.00E-02 |
| GOTERM_BP | peptidyl-tyrosine autophosphorylation | 5 | 4.00E-02 |
| GOTERM_BP | positive regulation of cellular biosynthetic process | 24 | 4.00E-02 |
| GOTERM_BP | positive regulation of developmental process | 19 | 4.10E-02 |
| GOTERM_BP | positive regulation of cyclic nucleotide biosynthetic process | 5 | 4.30E-02 |
| GOTERM_BP | antigen receptor-mediated signaling pathway | 6 | 4.40E-02 |
| GOTERM_BP | granulocyte differentiation | 4 | 4.80E-02 |
| GOTERM_BP | regulation of cyclic nucleotide metabolic process | 6 | 4.90E-02 |
| GOTERM_BP | regulation of lymphocyte proliferation | 7 | 4.90E-02 |
| GOTERM_BP | regulation of mononuclear cell proliferation | 7 | 4.90E-02 |
| GOTERM_BP | positive regulation of biosynthetic process | 24 | 4.90E-02 |
| GOTERM_BP | cellular response to chemical stimulus | 30 | 4.90E-02 |
| GOTERM_BP | positive regulation of purine nucleotide biosynthetic process | 5 | 4.90E-02 |
| GOTERM_BP | positive regulation of nucleotide biosynthetic process | 5 | 4.90E-02 |
| GOTERM_CC | plasma membrane part | 46 | 1.40E-07 |
| GOTERM_CC | plasma membrane | 58 | 5.70E-05 |
| GOTERM_CC | cell periphery | 59 | 5.70E-05 |
| GOTERM_CC | intrinsic component of plasma membrane | 26 | 2.70E-03 |
| GOTERM_CC | integral component of plasma membrane | 24 | 9.50E-03 |
| GOTERM_CC | plasma membrane receptor complex | 8 | 9.50E-03 |
| GOTERM_CC | membrane part | 75 | 9.50E-03 |
| GOTERM_CC | plasma membrane protein complex | 13 | 1.60E-02 |
| GOTERM_CC | side of membrane | 11 | 1.80E-02 |
| GOTERM_CC | extracellular space | 21 | 2.20E-02 |
| GOTERM_CC | membrane | 91 | 2.20E-02 |
| GOTERM_CC | intrinsic component of membrane | 63 | 4.10E-02 |
| GOTERM_CC | integral component of membrane | 62 | 4.60E-02 |
| GOTERM_CC | extrinsic component of cytoplasmic side of plasma membrane | 6 | 5.00E-02 |
| GOTERM_CC | cytoplasmic side of plasma membrane | 7 | 5.00E-02 |
| GOTERM_MF | receptor activity | 31 | 7.20E-05 |
| GOTERM_MF | molecular transducer activity | 31 | 7.20E-05 |
| GOTERM_MF | transmembrane receptor activity | 25 | 7.60E-04 |
| GOTERM_MF | receptor binding | 18 | 7.60E-04 |
| GOTERM_MF | transmembrane signaling receptor activity | 24 | 8.20E-04 |
| GOTERM_MF | signaling receptor activity | 25 | 1.30E-03 |
| GOTERM_MF | signal transducer activity | 28 | 2.10E-03 |
| GOTERM_MF | G-protein coupled receptor activity | 15 | 3.50E-02 |
| GOTERM_MF | cytokine receptor activity | 6 | 4.30E-02 |

**Supplementary Table S8. The DEGs enriched in the two KEGG pathways.**

| **Gene name** | **Description** | **Gene locus** | **log2FoldChange** | **FDR** |
| --- | --- | --- | --- | --- |
| Neuroactive ligand-receptor interaction | | | | |
| HTR1F | 5-hydroxytryptamine (serotonin) receptor 1F, G protein-coupled | 1:93982791-93983891 | 1.80 | 1.62E-02 |
| ADRA1D | adrenoceptor alpha 1D | 4:88341840-88377988 | 1.09 | 2.48E-02 |
| AVPR1A | arginine vasopressin receptor 1A | 1:33419044-33421641 | 1.38 | 1.90E-03 |
| C3AR1 | complement component 3a receptor 1 | 1:66793430-66796768 | -1.52 | 2.83E-02 |
| CRHR1 | corticotropin releasing hormone receptor 1 | 27:4646659-4670553 | 1.20 | 6.73E-03 |
| DRD4 | dopamine receptor D4 | 5:785916-791818 | 1.24 | 1.66E-02 |
| GRIA1 | glutamate receptor, ionotropic, AMPA 1 | 13:13030976-13139199 | 1.53 | 3.34E-05 |
| GRIK2 | glutamate receptor, ionotropic, kainate 2 | 3:70157498-70521359 | 1.39 | 1.60E-06 |
| GRIK3 | glutamate receptor, ionotropic, kainate 3 | 23:4000662-4069929 | 1.10 | 3.67E-02 |
| GZMA | granzyme A | Z:16643589-16666277 | -2.76 | 2.06E-04 |
| GHRHR | growth hormone releasing hormone receptor | 2:1238715-1257265 | -1.38 | 2.59E-02 |
| GH | growth hormone | 27:4034905-4038410 | -2.33 | 2.19E-03 |
| MC1R | melanocortin 1 receptor | 11:18840387-18841759 | -4.53 | 2.43E-03 |
| NPBWR2 | neuropeptides B/W receptor 2 | 20:9240792-9260447 | 1.07 | 2.81E-03 |
| Cytokine-cytokine receptor interaction | | | | |
| CCR2 | C-C motif chemokine receptor 2(CCR2) | 2:42632317-42640047 | -1.29 | 5.10E-03 |
| CCR5 | C-C motif chemokine receptor 5(CCR5) | 2:42617494-42623187 | -1.38 | 3.25E-02 |
| CCR8 | C-C motif chemokine receptor 8(CCR8) | 2:44316108-44319487 | -1.73 | 3.38E-02 |
| CX3CR1 | C-X3-C motif chemokine receptor 1(CX3CR1) | 2:44330678-44340445 | -1.50 | 1.60E-02 |
| FASLG | Fas ligand (TNF superfamily, member 6)(FASLG) | 8:4846877-4850856 | -2.14 | 2.17E-02 |
| TNFRSF13B | TNF receptor superfamily member 13B(TNFRSF13B) | 14:5052186-5055318 | -3.01 | 5.02E-03 |
| GH | growth hormone(GH) | 27:4034905-4038410 | -2.33 | 2.19E-03 |
| IL18RAP | interleukin 18 receptor accessory protein(IL18RAP) | 1:135229034-135244807 | -1.94 | 3.79E-02 |
| IL2RG | interleukin 2 receptor subunit gamma(IL2RG) | 4:2390550-2394261 | -1.84 | 3.84E-04 |
| IL20RA | interleukin 20 receptor subunit alpha(IL20RA) | 3:54956242-54977305 | -1.39 | 4.29E-02 |
